# Presence of leptin and its receptor in the ram reproductive system and *in vitro* effect of leptin on sperm quality

**DOI:** 10.7717/peerj.13982

**Published:** 2022-09-26

**Authors:** Yu Gao, Guodong Zhao, Yukun Song, Aerman Haire, Ailing Yang, Xi Zhao, Abulizi Wusiman

**Affiliations:** 1 College of Animal Science, Xinjiang Agriculture University, Urumqi, Xinjiang, China; 2 Department of Reproductive Medicine, Zhuozhou Hospital of Hebei Province, Zhuozhou, Hebei, China

**Keywords:** OB, OBR, Male reproductive system, Sperm parameters, Ram

## Abstract

Leptin is a 16 kDa hormone encoded by obese (*OB*) gene in adipocytes. This molecule not only regulates energy metabolism but also plays a role in the reproduction of mammals. Leptin and its receptor (*OBR*) have been found in male reproductive systems of human, bovine, equine and pig. The effects of leptin on sperm quality vary widely from different research findings. However, the presence of leptin and its receptor in the ram reproductive system and the *in vitro* effect of leptin on sperm quality have not reported yet. In the present study, we found that the *OB* was highly expressed in primary and secondary spermatocytes of the testes, *OBR* was highly expressed in secondary spermatocytes of the testes. The expressions of *OB* were in stereocilia of epididymis and in columnar cells of epididymal caput and cauda, the expressions of *OBR* were in columnar cells of epididymis and in stereocilia of epididymal *corpus* and cauda. The presence of both *OB* and *OBR* in testes, epididymis and sperm were confirmed through RT-PCR, immunolocalization and Western blot analyses. The RT-qPCR results indicated *OB* and *OBR* had higher expression levels in epididymal sperm than that of the ejaculated sperm in rams. When sperm were treated with 5 ng/mL leptin, the progressive motility (*P* < 0.01), straight-line velocity (VSL) (*P* < 0.05), average path velocity (VAP) (*P* < 0.05), membrane mitochondrial potential (MMP) (*P* < 0.01) and viability (*P* < 0.05) significantly increased, while DNA fragmentation index (DFI) and reactive oxygen species (ROS) significantly decreased compared to the control (*P* < 0.01), and the other semen parameters such as acrosome integrity and acrosome reaction rate had no significant changes between groups (*P* > 0.05). In conclusion, this is probably the first report describing localization of leptin and its receptors in the reproductive system of rams and their effects on sperm quality parameters. Our findings suggest that 5 ng/mL leptin treatment enhanced sperm motility, viability and MMP, and decrease DFI and ROS without obvious influence on the acrosome reaction in ram sperm. The potential mechanisms may be related to leptin’s ability to reduce the oxidative stress and apoptosis of sperms and improve their mitochondrial function and energy supply, therefore, to maintain the physiological homeostasis of the sperm.

## Introduction

Leptin is a 16 kDa polypeptide encoded by obese gene (*OB*) in adipocytes (Gonzalez et al., 2000). A small amount of leptin is also secreted by the anterior pituitary gland (Jin et al., 1999), ovary (Macedo et al., 2019), testis (Ishikawa et al., 2007), and placenta (Pazinato et al., 2019). Leptin acts by binding to the leptin receptor (*OBR*) and plays an important role in mammalian reproduction (Childs et al., 2021). Leptin receptors are widely distributed in the hypothalamus, pancreas, testis, and ovary and belong to the class 1 cytokine receptor family (Margetic et al., 2002). To date, six different leptin receptors have been identified (*OBRa-f*) and based on their structure defined as long, short, and soluble types (Malik, Durairajanayagam & Singh, 2019). After binding to its receptors, leptin can mediate multiple signaling pathways including Janus kinase 2 (JAK2)/signal transduction and activator of transcription 3 (STAT3), AMP-activated protein kinase (AMPK), mammalian target of rapamycin (mTOR), activated protein kinase phosphatase (MAPK) (Fruhbeck, 2006; Kwon, Kim & Kim, 2016), extracellular signal regulated kinase (ERK), and phosphate inositol kinase-3 (PI3K) pathways (Zhou & Rui, 2013).

As a pleiotropic molecule, leptin exhibits both positive and negative influences on sperm quality (Andò & Aquila, 2005; Leisegang et al., 2014; Xin et al., 2019). The evidence has indicated considerable differences as to the concentrations of leptin in seminal plasma of different species (Lackey, Gray & Henricks, 2002; Guo et al., 2014; Elfassy et al., 2018; El-Maaty et al., 2014; Kumar et al., 2016; SouzaI et al., 2012), the physiological implications of these differences in seminal plasma have not been clarified.

Leptin has been reported to exhibit a variety of physiological effects on the development of male gonads (Liu, Liu & Xu, 2020; Ohga & Matsuyama, 2021). For example, leptin is found to localize in seminiferous tubules and germ cells and its elevated levels are associated with spermatogenic dysfunction, potentially by jeopardizing the sperm motility (Glander et al., 2002). The *in vitro* treatment of sperm with leptin impacts several physological activities of sperm (Elfassy et al., 2018). When human sperm was treated with 10 μIU insulin and 10 nM leptin, the spermatozoon motility was significantly increased including the improvement of VCL and ALH, viability, acrosome reaction rate (Lampiao & du Plessis, 2008). On other hand, the incubation of sperm with leptin might not significantly influence their motility, acrosome reaction rate and sperm capacitation (Li et al., 2009). The expression of *OBR* has been detected on the plasma membrane of human sperm, indicating a potential involvement of leptin in male reproduction (Jope et al., 2003). Leptin and its receptor have also been found in the sperm of other animal species including pig (Aquila et al., 2008), bovine (Nikbakht et al., 2010; Abavisani et al., 2011) and horse (Lange-Consiglio et al., 2016).

The presence of leptin and leptin receptor in sperm might be linked with sperm capacitation and survival (Aquila et al., 2008). Leptin activates ERK1/2 which is involved in vital sperm maturation in the epididymis and sperm functions in the mature spermatozoa (Almog & Naor, 2010). In rat epididymis, ERK1/2 activation was also observed in the specific segment which is responsible for three growth factors including EGF, FGF2 and VEGFA, suggesting its involvement in sperm maturation (Tomsig, Usanovic & Turner, 2006). The presence of ERK1/2 in the post acrosomal region of human sperm suggests that ERK1/2 may participate in the acrosome reaction (Luconi et al., 1998a). Luconi et al. (1998b) found that ERK1/2 inhibitor PD098059 could block ERK activation during *in vitro* capacitation and also inhibited the acrosome reaction induced by progesterone. Almog et al. (2008) have reported that ERK1/2 is localized on the outer dense fibers and ERK1/2 involves in sperm motility. In addition, the binding of leptin with its receptor stimulates PI3K/Akt pathway. PI3K plays an important role in the survival of sperms. Collectively, these findings mentioned above indicated that leptin plays a crucial role sperm quality and survival (Almog et al., 2008). Based on these studies, we hypothesize that leptin may deeply involve in sperm physiology to improve sperm motility, viability, capacitation and acrosome reaction in rams. By literature search, we have found that several studies have reported the presence of leptin and leptin receptor in the ovary and their potential role in oocyte maturation (Kumar et al., 2019; Macedo et al., 2019; Menezes et al., 2019), however, few studies have dealt with the presence and potential implication of leptin and its receptor in ram reproductive system. Therefore, the present study was designed to investigate localization and expression of leptin and its receptor in the testes, epididymis, sperm in the Suffolk White rams and *in vitro* effect of leptin on their sperm quality.

## Materials and Methods

### Chemicals

In this study all chemicals were purchased from Sigma-Aldrich Co. (USA) unless stated otherwise.

### Animals

Suffolk White rams (*Ovis aries*) from Aoxin Animal Husbandry (Beijing, China), at the age of 1–2 years old with normal reproductive cycle, healthy and generally the similar body weight were selected for the experiments. All experimental protocols concerning the handling of animals were performed in accordance with the requirements of the Institutional Animal Care and Use Committee at the Xinjiang Agricultural University. The experiments were approved by the Animal Welfare and Animal Experimental Ethical Committee of Xinjiang Agricultural University (approval number 2020032).

### Experiment design

**Experiment 1:** The serum samples were collected at 08:00, 14:00, 18:00, and 24:00 h from jugular vein of six mature Suffolk White rams (1–2 years old), the seminal plasma was collected at 14:00 and 24:00 h of three mature Suffolk White rams (1–2 years old). The blood and seminal plasma were used to estimate the circadian changes of serum and seminal plasma levels of leptin in the Suffolk White rams.

**Experiment 2:** Testis and epididymal tissues were collected from three adult (1–2 years old) healthy Suffolk White rams, these tissues were used for immunohistochemical analysis to detect expression of leptin/leptin receptor genes. The artificial vagina method was used to collect the semen from eight mature robust Suffolk White rams (1–2 years old). For collection of sperm from the epididymis, the whole epididymis of three adult Suffolk White rams were collected. The ejaculated and epididymal sperm of the Suffolk White rams were used for RT-PCR, RT-qPCR, WB to determine the presence of *OB* and *OBR* in ram testis, epididymis and sperm including mRNA expression, protein expression, and immunolocalization.

**Experiment 3:** The semen was collected from eight matured robust Suffolk White rams (1–2 years old). Evaluation of spermatozoa quality included motility parameters (progressive motility, VSL, VAP), mitochondrial membrane potential (MMP), viability, DNA fragmentation index (DFI), reactive oxygen species (ROS), acrosome integrity and acrosome reaction rate. Based on pilot study, we confirmed that supplementation of Leptin with 5 ng/mL for 2 h *in vitro* is the best concentration and treatment duration.

### Testis and epididymal tissue collection

Testis and epididymal tissues were collected at Aoxin Animal Husbandry (Beijing, China) from three adult (1–2 years old) healthy Suffolk White rams.

The samples from castrated rams were immediately washed in phosphate-buffered saline (PBS) supplemented with antibiotics (penicillin, 100 IU/mL and streptomycin, 100 μg/mL). The tissues for histological analyses were incubated in fixative solution (4% paraformaldehyde) and other specimens were stored in liquid nitrogen for RT-PCR, Immunohistochemistry and Western blot (WB) analyses.

### Sperm selection and preparation

The artificial vagina method was used to collect the semen from eight matured robust Suffolk White rams (1–2 years old). Semen analysis was performed at 37 °C: the observed sperm concentration and motility were >2 × 10^9^/mL and >80%, respectively. The semen was transferred to tubes and centrifuged at 400*g* for 4 min. The upper layer of semen was used for leptin measurement and the lower layer of semen was purified by the swim-up method. Briefly, 0.2 mL of semen was deposited at the bottom of the tubes and then IVF medium (G-IVF plus; Vitrolife, Gothenburg, Sweden) was added up to 1 mL. The tubes were placed at 37 °C for 40 min under 5.0% CO_2_. The upper layer containing the mobile sperm was used for RT-PCR, RT-qPCR, WB, immunofluorescence and sperm parameters analyses.

For collection of sperm from the epididymis, the whole epididymis of three adult Suffolk White rams were sampled at Aoxin Animal Husbandry (Beijing, China) and transported to the lab with ice box. After making an incision in the epididymal, the content was mixed with IVF medium and centrifuged at 400*g* for 4 min. The analysis of sperm quality and the swim-up procedure were the same as above for the ejaculated sperm.

### Estimation of serum and seminal leptin

The serum samples were collected at 08:00, 14:00, 18:00, and 24:00 h from jugular vein and the seminal plasma were collected at 14:00 and 24:00 h of six mature Suffolk White rams (1–2 years old). The concentration of leptin was measured by radioimmunoassay using Leptin Direct RIA TM Kit (RE54021; Immuno-Biological Laboratories, Hamburg, Germany) followed the manufcture’s instructions.

### Histological analysis of testis and epididymal tissue

Paraffin sections were dewaxed with xylene, dehydrated with gradient alcohol, and then placed in distilled water. The samples were stained with hematoxylin solution (Servicebio, Wuhan, China) for 5 min. After washing with distilled water for 3 times, using 1% hydrochloric acid alcohol for color separation for 20 s. followed by incubation in 0.1M PBS pH7.4 for 3 min, The slices were washed through a graded alcohol concentrations for 2 min sequentially and stained with eosin (Servicebio, Wuhan, China) for 30 s. Finally, the samples were detained in 95% alcohol for 1 min, dehydrated in anhydrous ethanol and xylene.

### Immunohistochemical analysis for expression of leptin/leptin receptor

The testis and epididymal tissues were fixed by paraffin-embedded (FFPE) method as described in section above. The paraffin slides were dewaxed with xylene, dehydrated with gradient concentrations of ethanol process, washed with deionized water, and placed in 3% H_2_O_2_ for 10 min. Thereafter, the samples were blocked with 1% Goat serum in PBS for 20 min. subsequently, the samples were incubated with the primary antibody rabbit anti-sheep *OB* antibody (1:100, ab3583; Abcam, Cambridge, UK) or rabbit anti-human *OBR* antibody (1:100, 20966-1-AP; Proteintech, Rosemont, IL, USA) in a humidified chamber at 4 °C, overnight. Then, the samples were incubated with the secondary antibodies (goat anti-rabbit IgG) at a dilution of 1:1,000 (Abcam, Cambridge, UK) for 60 min and then reacted with 3,3′-diaminobenzidine (DAB) as chromogen substrate (Servicebio, Wuhan, China). The negative controls (NG) were processed identically as above, except for primary antibody being IgG. The expression of *OB* and *OBR* in immunohistochemical staining was evaluated using ImageJ software analysis color intensity with relative grey value. Immunohistochemical analysis was repeated at least three times for each experiment.

### Immunofluorescence detection of leptin and leptin receptor

Fresh ejaculated sperm were selected by the swim-up procedure, centrifuged, and fixed for 10 min with 4% paraformaldehyde solution. After the removal fixative solution, sperms were washed in TBSTx (0.1% Triton X-100 in TBS) and placed in a blocking solution (5% BSA in TBSTx) for 1 hour. After blocking, the specimens were incubated with primary antibodies *OB* (1:100, ab3583; Abcam, Cambridge, UK) and *OBR* (1:100, 20966-1-AP, Proteintech, Rosemont, IL, USA) antibodies at 4 °C, overnight in a humidified box. For the control sperm slides, normal rabbit serum was used. Sperms were then washed (centrifuging at 300*g* for 2 min and resuspending in PBS) four times and incubated for 2 h with the secondary antibody (Alexa Fluor488, P0176; Beyotime Institute of Biotechnology, Shanghai, China). After washing two times, the slides were coated with 10 μL concentrated sperm suspension and covered with a coverslip. The slides were analyzed by a confocal microscope (SP8; Leica, Richmond, IL, USA). Negative controls (NG) were were processed identically as above, except for the lack of primary antibodies. PBS was used in its amount instead of the primary antibody. Immunofluorescence analysis was repeated at least three times for each experiment.

### Analysis of mRNA expression of *OB* and *OBR* using reverse-transcription PCR and quantitative RT-PCR

The total RNA was extracted from testis, epididymal tissue, adipose tissue, ejaculated and epididymal sperms with Trizol LS extraction kit and Turbo DNA-free kit (Ambion, Austin, TX, USA) using a standard protocol. The applied PCR primers and the expected amplification product lengths were shown in Table 1. RT-PCR procedures were performed as per the manufacturers’ guidelines (Cells-to-cDNA TM Kit, AM1722; Ambion Company, Austin, TX, USA). The PCR conditions were as follows: initial denaturation at 95 °C for 10 min, 35 denaturation cycles at 94 °C for 30 s, annealing at Tm for 45 s, and then extension at 72 °C for 15 s, followed by final extension at 72 °C for 10 min. The levels of relevant mRNAs, including the products of *OB* and *OBR* in epididymal and ejaculated sperm were quantified by real-time qPCR using One-Step SYBR RT-PCR Kit (Takara, Japan) in a light cycler (Roche Applied Science, Penzberg, Germany). The qPCR program included a 10 min incubation at 95 °C to activate FastStart DNA polymerase, followed by 35 cycles of 95 °C for 10 s, 60 °C for 15 s and 72 °C for the appropriate extension time with single fluorescence acquisition. Each sample was run in triplicate and the mean cycle threshold (Ct) value was transformed to relative expression level by the 2^−ΔΔCt^ method (Livak & Schmittgen, 2001). *GADPH* was used as an internal control for normalization.

**Table 1:** Details of primers used in the present study.

Genes	Accession ID	Primer sequence (5′-3′)	Tm (°C)	Product size (bp)
*GAPDH*	NM_001190390.1	Forward: TCGGAGTGAACGGATTTG	54.3	173
		Reverse: CTCTGCCTTGACTGTGCC	53.7	
*OB*	XM_027968780.2	Forward: ACAGAGGGTCACTGGTTTG	52.1	172
		Reverse: CAGCAGGTGGAGAAGGTC	52.5	
*OBR*	NM_001009763.1	Forward: AGTATTTACGGAAGGAGT	42.9	122
		Reverse: AGATTGAGGAGGAGATTA	42.6	

### Western blot analysis for *OB* and *OBR* protein expression

Western blot was used for analysis of *OB* and *OBR* proteins in testis, epididymal tissue, ejaculated and epididymal sperm of rams as described by the method of Zhang (Zhang et al., 2017). Briefly, tissues or cells were lysed with a lysis buffer (HX1862; Huaxingbo, Hong Kong, China). Equal amounts of protein were resolved using 12% SDS-PAGE gel and transferred to PVDF membranes (ISEQ00010; Millipore, Burlington, MA, USA). After they were blocked with 5% nonfat milk or BSA (A3311; Sigma-Aldrich, St. Louis, MI, USA) at 37 °C for 60 min, the membranes were incubated with primary antibodies against *OB* (1:1,000, ab3583, Abcam, Cambridge, UK), *OBR* (1:1,000, 20966-1-AP, Proteintech, Rosemont, IL, USA) and GAPDH ( 1:2,500, WL01114; Wanleibio, Hebei, China) at 4 °C, overnight. The membranes were then washed 3–5 times with TBST buffer (HX1893; Huaxingbo, Hong Kong, China) and incubated with horseradish peroxidase (HRP)-conjugated secondary antibodies (1:5,000, 7074; Cell Signaling Technology, Danvers, MA, USA). Western blotting detection reagents (GE Healthcare, Uppsala, Sweden), and then were visualized using a cooled CCD camera (Nikon, Tokyo, Japan) based chemiluminescence detector (GE Healthcare, Uppsala, Sweden). Protein bands were then analyzed by computer software (Image-Quant 350; GE Healthcare, Uppsala, Sweden).

### Spermatozoa motility parameters

The upper layer containing the mobile sperm as described in section (Sperm selection and preparation) placed in Disposable Sperm Counting Plate (SAS, Beijing, China), The concentration of sperm and motility was measured by computer-assisted sperm analyzer (CASA, SAS, Beijing, China).

### Spermatozoa viability (Plasma Membrane Integrity)

The Eosin–Nigrosin test (SAS, Beijing, China) was used to assess membrane integrity of sperm for only one-step staining. The nigrosine created a black background, making stained sperm easier to distinguish. The samples were observed with a 400× phase microscope (CX41; Olympus, Tokyo, Japan), the head of live sperm was white or light pink, the head of dead sperm was red or dark pink.

### Mitochondrial membrane potential (MMP)

MMP were assessed with a JC-1 Assay Kit (SAS, Beijing, China) based on the manufacturer’s instruction. JC-1 emission fluorescence changed from red (~595 nm) to green (~535 nm) as the MMP changes from aggregates to monomers. sperm was diluted to 500 μL, 1–2 × 10^6^ /ml in PBS, added to 500 μL of JC-1 staining working solution, incubated at 37 °C for 15 min in the dark, centrifuged at 300*g* for 5 min, and the supernatant was discarded, Resuspend in 1.0 ml of JC-1 staining buffer (1×), centrifuged at 300*g* for 5 min, discard the supernatant, add 300 μL of JC-1 staining buffer (1×) to resuspend, Finally, the samples were detected by flow cytometer (LSR Fortessa, BD, USA).

### Sperm chromatin dispersion test (SCD)

The SCD test is assessed with a SCD Assay Kit (SAS, Beijing, China) based on this principle that when the head of spermatozoon lysed, intact DNA loops of sperm expanded, but fragmented DNA of sperm does not expand or is minimally expanded. The semen was diluted in DPBS to a concentration of 5 × 10^6^/ml and 60 μL was added to Eppendorf tubes containing low-melting point aqueous agarose. The sperm suspension in the agarose was mixed well, Aliquots of 30 μL suspension were placed onto a agarose precoated glass slice, The slice was placed on a cold surface at 4 °C for 5 min, The coverslip was gently removed immersed in lysis solution I for 7 min, placed in lysis solution II for 25 min, washed in distilled water, the slide was dehydrated in gradient ethanol (70%, 90%, 100%) for 2 min each, let the slide to dry in an oven at 37 °C, a layer of dye solution was covered for 15 min and the slide was washed in tap water, then air dry, the sperm was assessed under a light microscope (Olympus CX41; Olympus, Tokyo, Japan) at 400-fold magnification.

### Sperm reactive oxygen species (ROS)

Intracellular ROS of sperm was assessed with a ROS Assay Kit (SAS, Beijing, China) containing the 2′,7′-dichlorodihydrofluorescein diacetate (H2DCFDA) according to the manufacturer’s instruction. In brief, 1 µL H2DCFDA and 5 µL PI were added into the sperm suspension and mixed well, incubated at 37 °C for 30 min in the dark. the samples were centrifuged, suspended with PBS. Then, the samples were detected by flow cytometer.

### Sperm acrosome integrity (AI) and acrosome reaction (AR)

AI and AR of sperm are measured using a AR Assay Kit (SAS, Beijing, China) containing *Pisum sativum* agglutinin (PSA) labelled with fluorescein isothiocyanate (FITC) (PSA-FITC). Briefly, the control tube contains 0 ng/mL leptin and the test tube contains 5 ng/mL leptin incubated in Biggers-Whitten-Whittingham (BWW) medium supplemented with bovine serum albumin (Solarbio, Beijing, China). The samples were incubated at 37 °C, 5.0% CO_2_, for 3 h in air incubator. The samples were stained with 1.5 µL PI and 2.5 µL PSA-FITC for 10 min in the dark, centrifuged, suspended with PBS and analyzed by flow cytometer. Acrosome Integrity (AI%) was defined as the percentage (%) of FITC-PSA-positive sperms, the inducibility of acrosome reaction (AR%) was defined as the percentage (%) of FITC-PSA-negative sperms having undergone acrosome reaction.

### Statistical analysis

Data are expressed as mean ± SD. Statistical analyses were performed using one-way analysis of variance (ANOVA) followed by student *t* test using SPSS 21.0 statistical software. *P < 0.05 was considered statistically significance. **P < 0.01 was considered highly significant difference. At least three independent experiments were repeated for each finding.

## Results

### The circadian profile of serum and seminal plasma levels of leptin in the Suffolk White rams

**Figure 1:**
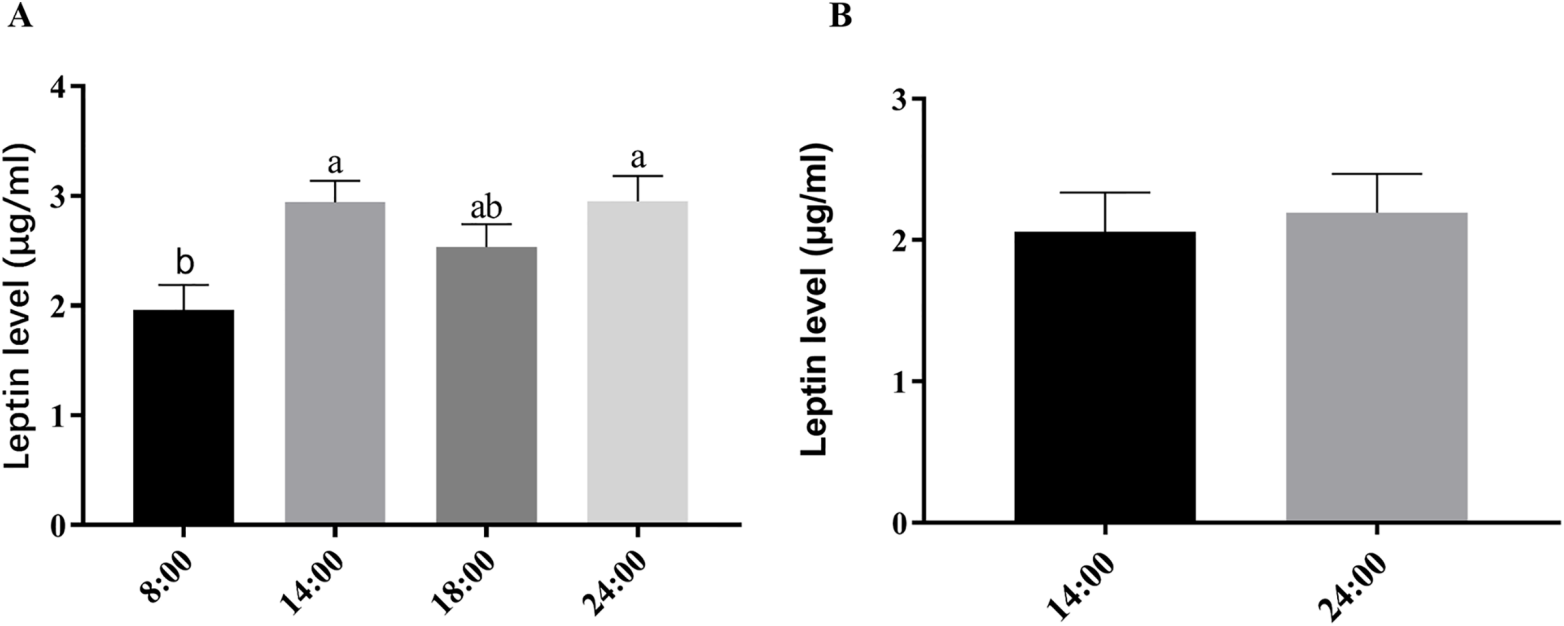
Comparison of serum and seminal plasma leptin levels at different time-points in the Suffolk White rams. Serum leptin levels at different time-points (A), the bars represent mean ± SD (*n* = 6). Seminal plasma leptin levels at different time-points (B), the bars represent mean ± SD (*n* = 3). Different letters in the same column indicate significant difference (*P* < 0.05).

The concentrations of serum leptin in the Suffolk White rams were not show an obvious circadian rhythm even the highest level was detected at the 24:00 h (Fig. 1A). There were no significant differences in the leptin levels in the seminal plasma of the Suffolk White rams between two time points of 14:00 and 24:00 (*P* = 0.748) (Fig. 1B).

### Expression of *OB* and *OBR* in testis, epididymis, and sperm of the Suffolk White rams

**Figure 2:**
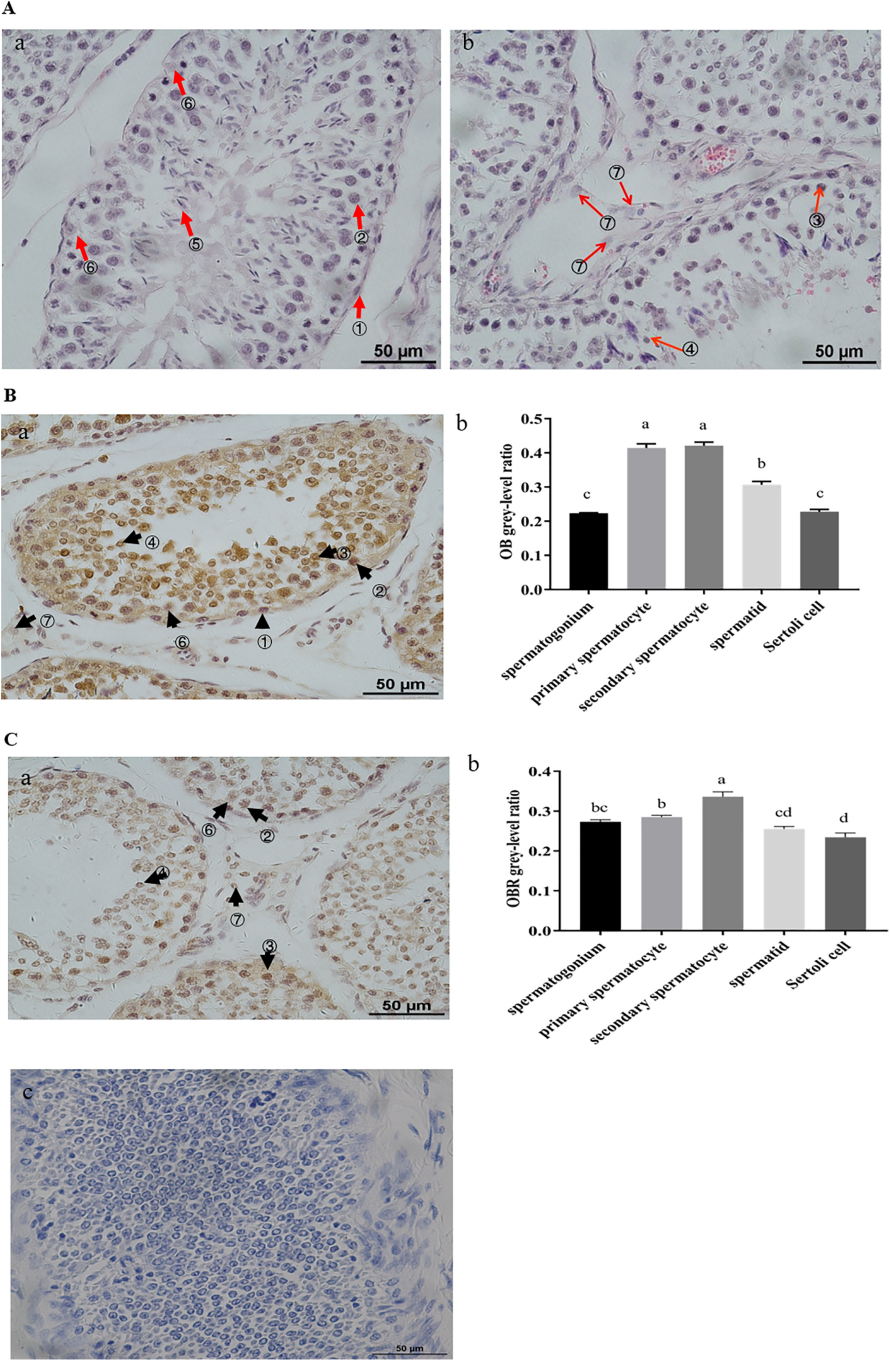
Expression of *OB* and *OBR* in ram testis. (A) HE staining in ram testis. (B) *OB* protein distribution and grey-level analysis in ram testis, (Ba) *OB* protein distribution in ram testis, (Bb) *OB* protein grey-level analysis in ram testis. (C) *OBR* protein distribution and grey-level analysis in ram testis, (Ca) *OBR* protein distribution in ram testis, (Cb) *OBR* protein grey-level analysis in ram testis, (Cc) Negative controls (NG) were identical, except for primary antibody is IgG. The arrows indicate: ① spermatogonium, ② primary spermatocyte, ③ secondary spermatocyte, ④ spermatid, ⑤ spermatozoon, ⑥ Sertoli cell, and ⑦ Leydig cell. Scale bar: 50 μM. Different letters in the same column indicate significant difference (*P* < 0.05). Scale bar: 50 μM.

In the testis, the *OB* expression was observed in spermatogonium, primary spermatocytes, secondary spermatocytes, spermatids, and Sertoli cells (Fig. 2B). Surprisingly, the primary and secondary spermatocytes showed the highest expression *OB* (*P* < 0.01) (Fig. 2Bb). Also, *OBR* expression has been identified in spermatogonium, primary and secondary spermatocytes, spermatids, and Sertoli cells (Fig. 2C). The strongest expression of *OBR* was observed in secondary spermatocytes (*P* < 0.05) (Fig. 2Cb).

**Figure 3:**
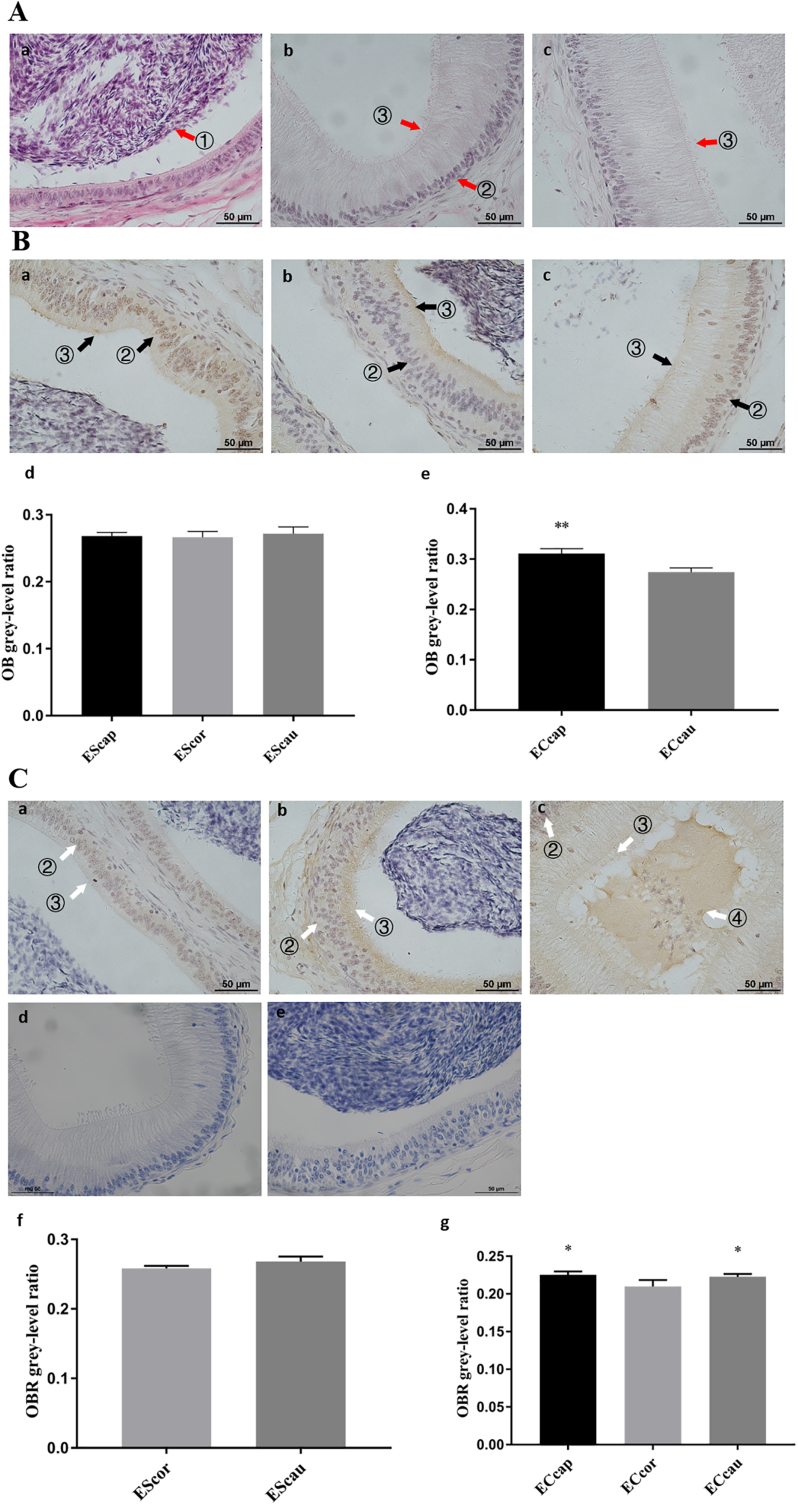
Expression of *OB* and *OBR* in ram epididymis. (A) HE staining in ram epididymis. (B) *OB* protein distribution and gray-level analysis in ram epididymis, (Ba) *OB* protein distribution in epididymal caput, (Bb) *OB* protein distribution in epididymis *corpus*, (Bc) *OB* protein distribution in epididymal cauda, (Bd) *OB* protein gray-level analysis in stereocilia of epididymis, (Be) *OB* protein gray-level analysis in columnar cells of epididymis. (C) *OBR* protein distribution and gray-level analysis in ram epididymis, (Ca) *OBR* protein distribution in epididymal caput, (Cb) *OBR* protein distribution in epididymis *corpus*, (Cc) *OBR* protein distribution in epididymal cauda, (Cd, Ce) Negative controls (NG) were identical, except for primary antibody is IgG, (Cf) *OBR* protein gray-level analysis in stereocilia of epididymis, (Cg) *OB* protein gray-level analysis in columnar cells of epididymis. ① epididymal spermatozoon, ② columnar cells, ③ stereocilia cells, and ④ soluble *OBR* isoforms. EScap: stereocilia of epididymal caput, EScor: stereocilia of epididymal *corpus*, EScau: stereocilia of epididymal cauda, ECcap: columnar cells of epididymal caput, ECcor: columnar cells of epididymal *corpus*, ECcau: columnar cells of epididymal cauda. Scale bar: 50 μM. Gray-level ratio of *OB* and *OBR* in ram epididymis (B–E). The bars represent mean ± SD. *P < 0.05 was considered to be significantly different. **P < 0.01 was considered to be highly significantly different.

In the epididymis, it was observed that *OB* was expressed in the stereocilia of the epididymis (Fig. 3Bd), while *OBR* was expressed in the stereocilia of the epididymal *corpus* and epididymal cauda (Fig. 3Cf). *OB* expression in columnar cells of epididymal caput was stronger than that in columnar cells of epididymal cauda (*P* = 0.007), while no signals has been detected in columnar cells of epididymal *corpus* tissues (Fig. 3B). *OBR* expression was detected in columnar cells of epididymis (Fig. 3C). The soluble *OBR* expression was also found in the epididymal lumen (Fig. 3Cc).

### Immunolocalization of *OB* and *OBR* in sperm of the Suffolk White rams

**Figure 4:**
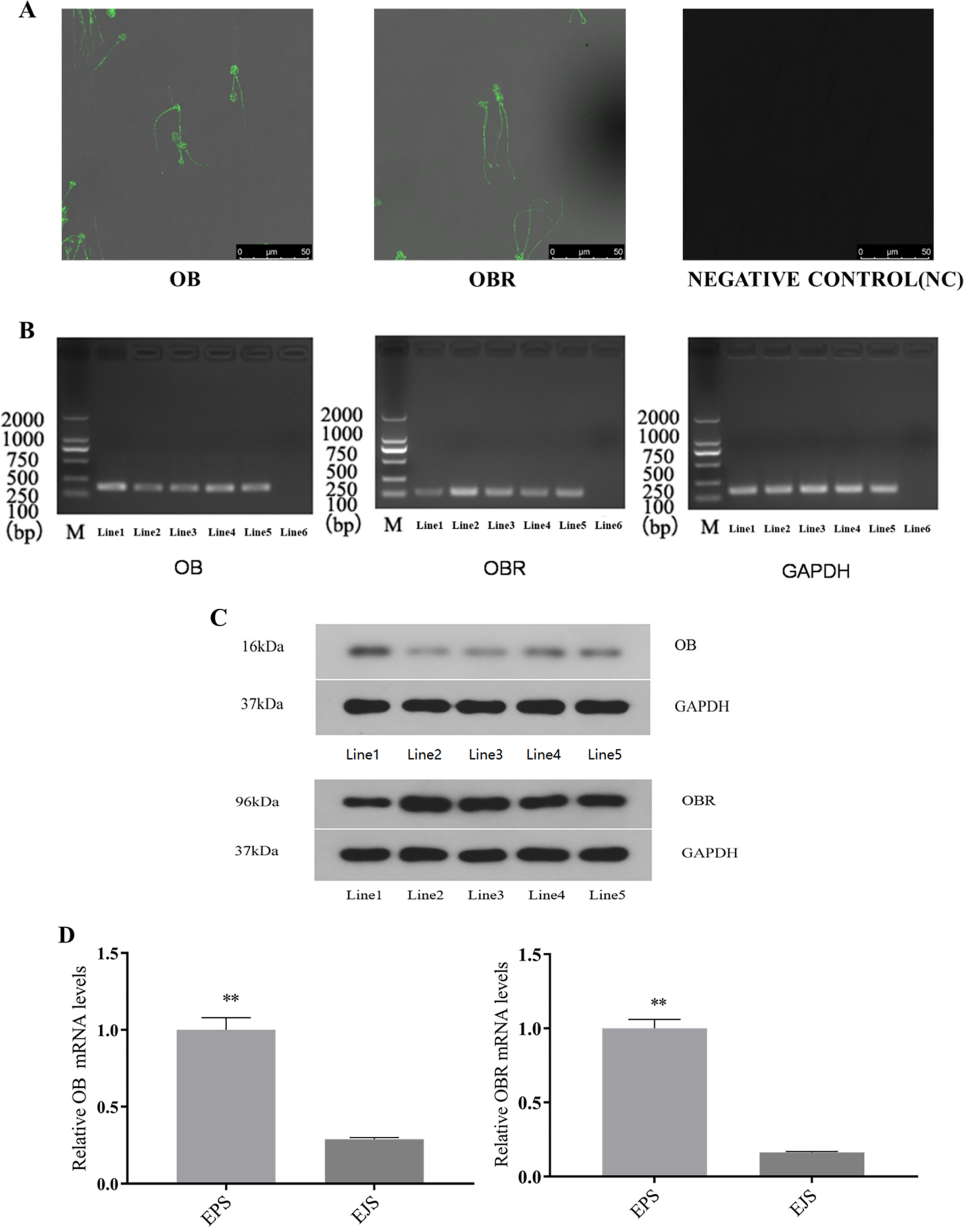
Expression of *OB* and *OBR* in the ram reproductive system and spermatozoa. Representative immunofluorescence labeling of *OB* and OBR in ram sperm (A). Immunolocalization of *OB* and *OBR* in the sperm head and tail region. All samples were incubated with goat anti-rabbit as negative controls. Scale bar: 50 μM. mRNA expression *OB* and *OBR* in ram sperm (B). Signal for *GAPDH* (employed as a reference gene) and leptin transcripts observed in: Line1, testicular tissue, Line2, epididymal tissue, Line3, ejaculated sperm, Line4, epididymal sperm, Line5, adipose tissue (positive control), and Line6, blank (negative control). Marker: 2,000 bp. *OB*, *OBR*, and *GAPDH* expression originated from the same gel. Western blot analysis of *OB* and *OBR* proteins in the tissues and sperm of Suffolk White ram (C). Line1, testis, Line2, epididymal tissue, Line3, ejaculated sperm, Line4, epididymal sperm and Line5, adipose tissue (positive control). *GAPDH* was used as a reference. The relative mRNA expression of *OB* and *OBR* in epididymal and ejaculated sperm in the Suffolk White rams (D). EPS, epididymal sperm; ELS, ejaculated sperm, ** *P* < 0.01 was considered to be highly significantly different.

The immunocytochemical assay revealed that both *OB* and *OBR* were expressed in the whole sperm including the head and tail (the middle piece, principal piece, and end piece) (Fig. 4A).

### Detection of *OB* and *OBR* in testis, epididymal tissue, ejaculated and epididymal sperm with RT-PCR

The RT-PCR analysis identified mRNA expression of both *OB* and *OBR* in testis, epididymal tissue, ejaculated and epididymal sperm (Fig. 4B). This finding was supported by the WB analysis which confirmed the presence of that the proteins of both *OB* and *OBR* in testis, epididymal tissue, ejaculated and epididymal sperm. The leptin protein of the Suffolk White rams was recorded as 16 kDa and that of leptin receptor was observed to be 96 kDa (Fig. 4C). The RT-qPCR results indicated *OB* and *OBR* gene expression levels in epididymal sperm were significantly higher (*P* < 0.01) than that in the ejaculated sperm (Fig. 4D).

### Effect of leptin supplementation on Spermatozoon motility, MMP and viability

**Figure 5:**
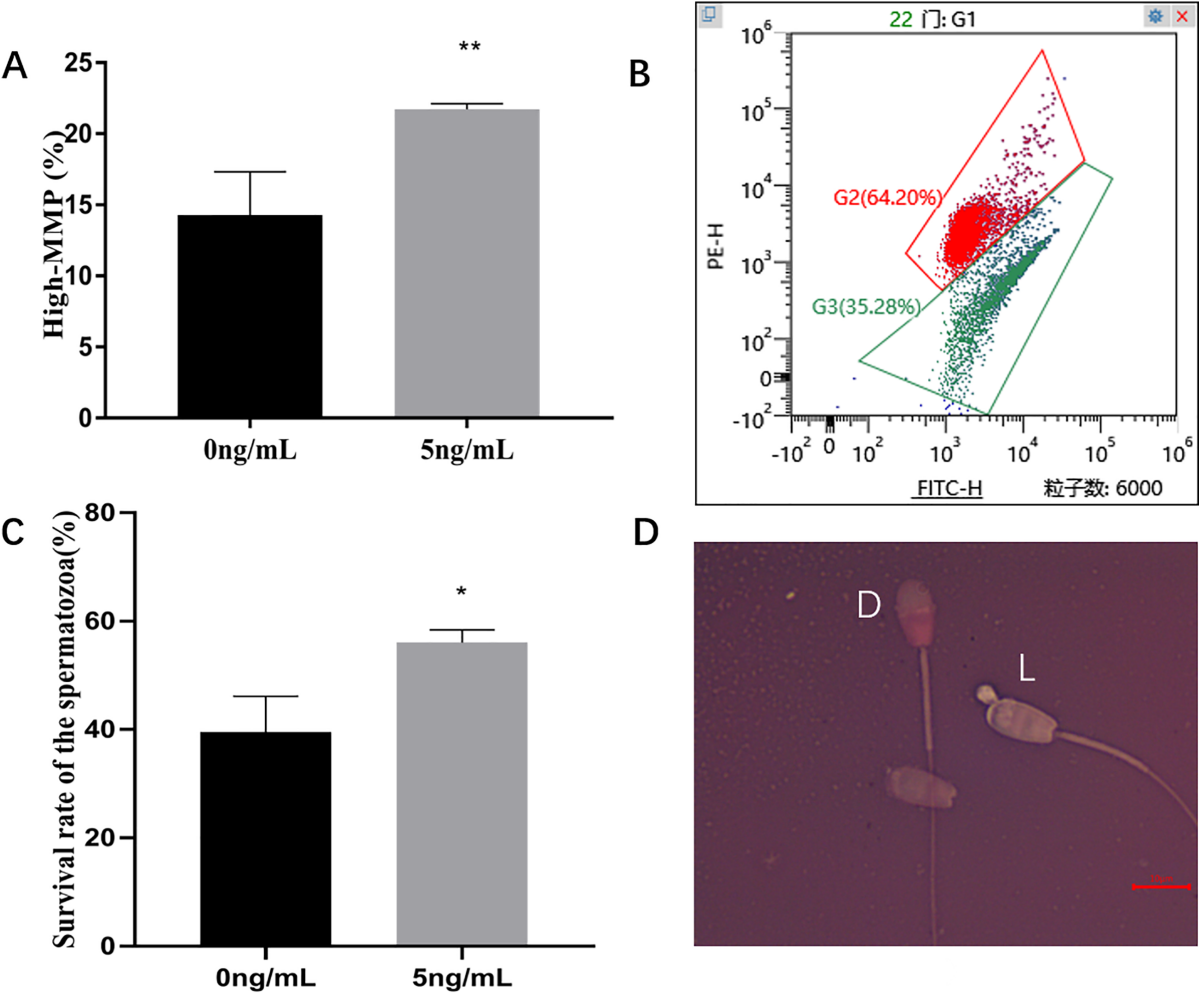
Effects of leptin on MMP and survival rate of sperm in rams. (A) Effects of 5 ng/mL leptin on high-MMP rate of sperm. (B) Flow cytometry evaluation of MMP, Red (I) represents high-MMP, Green (II) represents low-MMP. (C) Effects of 5 ng/mL leptin on survival rate of sperm. (D) Eosin-nigrosin staining for sperm (Scale bar: 10 μM). Sperm with red or dark pink head (D) is considered dead while sperm with white or light pink heads (L) is considered alive. *P < 0.05 was considered to be significantly different, **P < 0.01 was considered to be highly significantly different.

Incubation of sperms with leptin 5 ng/mL for 2 h, compared with the control group (0 ng/mL), significantly improved the sperm progressive motility (*P* = 0.005), VSL (*P* = 0.014), VAP (*P* = 0.015) compared to the control group (Table 2). JC-1 staining showed that, 5 ng/mL Leptin- treated group significantly increased MMP of ram sperm compared to the control (*P* = 0.003) (Figs. 5A, 5B). Eosin-nigrosine staining showed that 5 ng/mL Leptin-treatment was significantly increased the survival rate of the sperms compared to the control. (*P* = 0.015) (Figs. 5C, 5D).

**Table 2:** Ram sperm motility parameters in different concentrations of leptin were treated *in vitro* for 2 h.

Leptin	Progressive motility (%)	VSL (μ m s^−1^)	VAP (μ m s^−1^)	LIN (%)	ALH (μ m)	STR (%)
0 ng/mL	49.89 ± 3.93	48.61 ± 3.24	58.31 ± 3.42	50.06 ± 0.37	2.66 ± 0.01	83.35 ± 0.20
5 ng/mL	64.01 ± 1.78**	59.93 ± 3.42*	71.46 ± 3.90*	50.93 ± 1.08	2.69 ± 0.04	83.81 ± 0.59

**Notes:**

Data are expressed as mean ± SD.

* *P* < 0.05 was considered to be significantly different.

** *P* < 0.01 was considered to be highly significantly different. At least three independent experiments were repeated for each finding. VSL, straight-line velocity; VAP, average path velocity; AHL, average lateral head; STR, sttfnraightness.

### Effect of leptin on Spermatozoon DNA fragmentation index (DFI) and reactive oxygen species (ROS)

**Figure 6:**
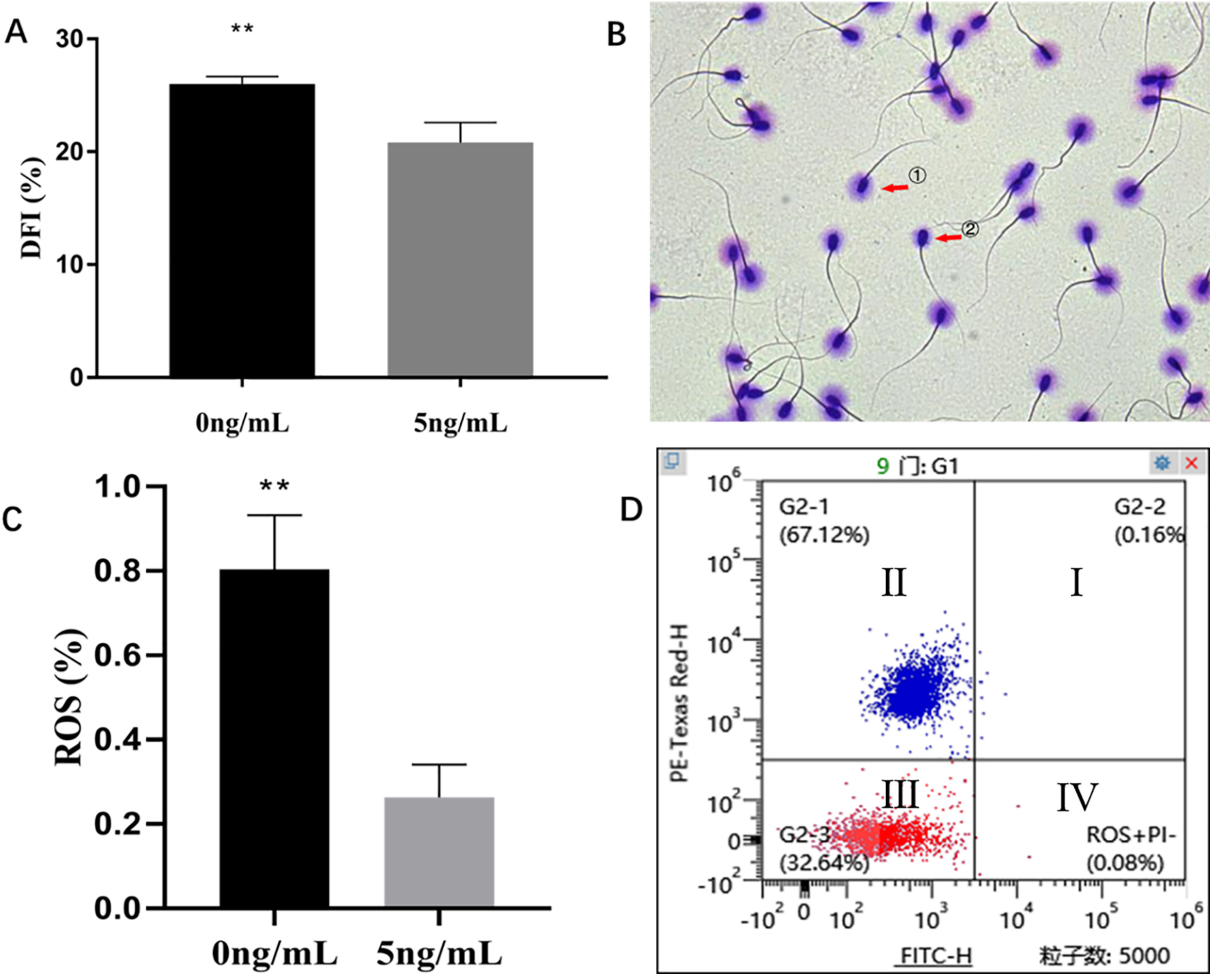
Effects of leptin on DFI and ROS of sperm in rams. (A) Effects of 5 ng/mL leptin on DFI rate of sperm. (B) Sperm Chromatin Dispersion Test for sperm (×400). sperm with (① intact DNA) and without halo (② fragmented DNA). (C) Effects of 5 ng/mL leptin on ROS rate of sperm, (D) Flow cytometry evaluation of ROS, Red represents alive sperm (III: ROS−/PI−, IV: ROS+/PI−), Blue (I, II) represents dead sperm. **P < 0.01 was considered to be highly significantly different.

Sperm Chromatin Dispersion Test (SCD) showed that leptin (5 ng/mL) treatment sperms had significantly lower DFI than that of control group (0 ng/mL) (*P* = 0.004) (Figs. 6A, 6B). The H_2_DCFDA test showed that leptin treated sperms had a significantly lower ROS than that of control (*P* = 0.0059) (Figs. 6C, 6D).

### Effect of leptin supplementation on Spermatozoon AI and AR rate

**Figure 7:**
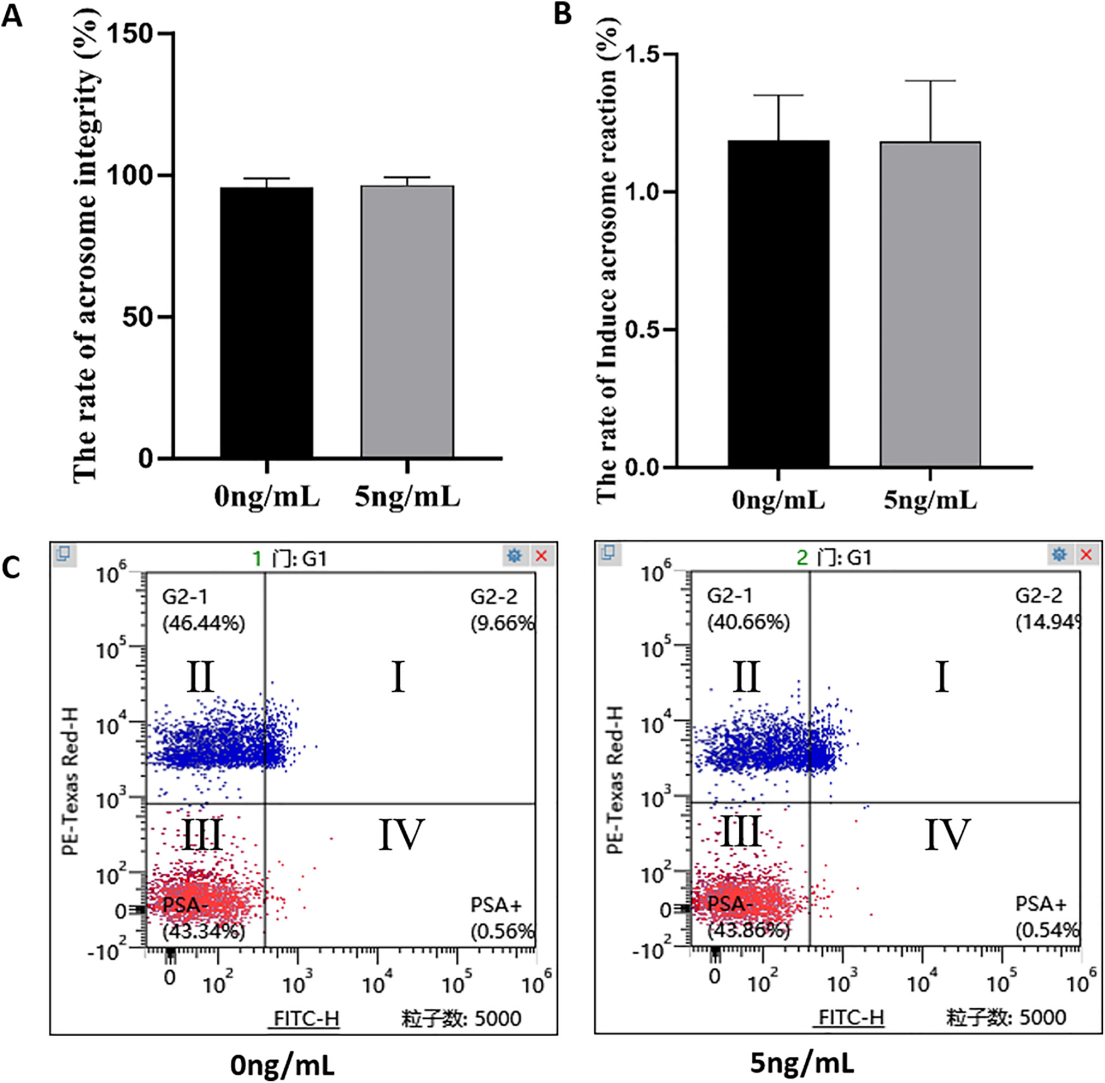
Effects of leptin on acrosome integrity and acrosome reaction rate of sperm in rams. (A) Effects of 5 ng/mL leptin on acrosome integrity rate of sperm. (B) Effects of 5 ng/mL leptin on acrosome reaction rate of sperm. (C) Flow cytometry evaluation of acrosome integrity and acrosome reaction of sperm, Red represents alive sperm (III: PSA−/PI−, IV: PSA+/PI−), Blue (I, II) represents dead sperm. *P* > 0.05 was considered to be no significantly different.

No significant difference was found in sperm acrosome integrity between leptin treated sperms and the controls (*P* = 0.852) (Figs. 7A, 7C) and the same was in spermatozoa between the groups. (*P* = 0.852) (Figs. 7B, 7C).

## Discussion

Leptin is an important molecule involved in regulation of mammalian energy metabolism and its action is mainly mediated by its central and local receptors. Leptin deficiency and mutation of its receptors are associated with various disorders including obesity, diabetes, heart disease and neurodegenerative diseases (Kang, Ok & Lee, 2020; Calio et al., 2021; Hontsariuk et al., 2020). In addition, leptin plays an important role in mammalian reproduction, particularly in males (Childs et al., 2021; Chang et al., 2021). For instance, the level of leptin in the semen of varicocele patients was significantly higher than that in the normal subjects. The seminal leptin content was positively correlated with varicocele classification and inversely proportional to sperm motility (Ishikawa et al., 2007; Chen et al., 2009; El Taieb et al., 2019). But the controversial case was also reported. For example, a study showed that leptin significantly increased human sperm motility, vitality, acrosome reaction, fertilization and nitric oxide (NO) (Lampiao & du Plessis, 2008). The relationship between seminal leptin concentration and sperm motility has not clearly established currently, however, it seems that the effects of leptin on male reproduction are concentration dependent in seminal plasma. At lower physiological concentrations, leptin promotes sperm quality while at high concentration it reduces sperm quality (Khaki, Batavani & Najafi, 2013).

Given that the biological effects of leptin are mediated by leptin receptors in target tissues, we examined the immunolocalization of both *OB* and *OBR* in testis and epididymal tissues. By use of the technology of molecular biology, we have confirmed for the first time that both mRNA and proteins of *OB* and *OBR* are expressed in the reproductive system of the Suffolk White rams. The *OB* and *OBR* were widely distributed in the seminiferous tubules of testis, primary spermatocytes, secondary spermatocytes, spermatids, and Sertoli cells. Their highest expressions were found in the primary and secondary spermatocytes. Our findings were consistent with the report leptin was expressed on germ cells, especially in spermatocytes (Ishikawa et al., 2007). Previously, the mRNA of *OBR* was mainly found in Leydig and Sertoli cells of rats (Tena-Sempere et al., 2001). In humans, leptin was only detected in seminiferous tubules (Glander et al., 2002). However, Ishikawa et al. (2007) found that leptin was present in both germ cells and seminiferous tubules, while leptin receptor was expressed in Leydig cells. In pigs, leptin and leptin receptor were only detected in seminiferous tubules and Leydig cells (Rago et al., 2009). In dogs, *OB* and *OBR* were weakly and sporadically expressed in Leydig cell, Sertoli cells, spermatocytes and spermatids (Müller et al., 2017). In comparison to other species, our results revealed species-specific expression of *OB* and *OBR* in reproductive system of the Suffolk White rams, indicating that leptin and its receptor may have species-specific function as well.

It was reported that leptin stimulates germ cells proliferation and differentiation in testis (Müller et al., 2017; Herrid, O’Shea & McFarlane, 2008). The expression of *OB* and *OBR* in Leydig cells may directly relate to the autocrine and paracrine roles of leptin on the production of sex steroids in these cells. This was supported by the previous report in which leptin induced the STAT3 signaling pathway in isolated seminiferous tubules to promote sex steroids production and regulate the regeneration and differentiation of spermatogenic cells (El-Hefnawy, Ioffe & Dym, 2000). The effects of leptin on synthesis of multiple sex steroids have also been observed in mammalian ovaries (Lv et al., 2019). In addition to expression of *OBR* in the stereocilia of the epididymal *corpus* and epididymal cauda, the soluble *OBR* was also found in the epididymal lumen. The presence of soluble *OBR* isoform in the epididymis fluid has not been identified in other species.

The *OBR* exists in multiple isoforms in rodents, a soluble isoform is produced by alternative splicing. As the membrane hydrolyzed by proteases, the *OBR* of membrane formed soluble receptor (Maamra et al., 2001). But the mRNA encoding soluble receptor has not been reported in human to date. Evidence from cultured cells shows that soluble receptors of leptin as antagonists of the leptin, inhibits the surface binding and internalization of leptin (Tu et al., 2008). The changes in leptin sensitivity are associated with alterations in the concentrations of its serum soluble receptor. For example, the increased level of soluble receptor may inhibit the action of leptin directly while the decreased amounts of soluble receptor seem to reflect reduced expression of *OBR* on membrane (Schaab & Kratzsch, 2015). Interestingly, our results showed that during spermiogenesis and epididymal transit, the expression of *OB* and *OBR* in epididymal spermatozoon was significantly higher than that in ejaculated spermatozoon, indicating the importance of *OB* and *OBR* on sperm maturation. Myles et al. (1987) believed that the surface of sperm had dynamic proteins that were frequently changed. The altered expression of the surface proteins (localization and expression levels) may affect sperm function post-testicular. Based on this finding, we speculate that the signal transduction pathway of leptin might be involved in modulation of sperm maturity in the epididymis. However, further focused research is needed to reinforce this notion and improve our understanding in this regard.

This study also provides novel evidence regarding the presence of *OB* and *OBR* proteins in ram sperm. We have identified that the molecular weight of *OBR* isoform in the Suffolk White ram was 96 kDa which differs from the *OBRs* found in pig sperm, the six *OBR* isoforms found in pigs are 120, 90, 80, 65, 60, and 40 kDa, respectively (Aquila et al., 2008). The *OBR* isoform found in horse is 90 kDa (Lange-Consiglio et al., 2016). In addition, the distribution of *OB* and *OBR* showed the species-specific pattern in sperm. In ram, the *OB* and *OBR* were expressed throughout the sperm which is similar to the boar sperm (De Ambrogi et al., 2007), but its expression was different from other species in which *OBR* was only located in the tail piece of human sperm (Jope et al., 2003), otherwise, the *OBR* was located on the acrosome of boar sperm (Aquila et al., 2008) and the *OB* and *OBR* were weakly detected in the middle of equine sperm and in the post-acrosomal area (Lange-Consiglio et al., 2016).

Our results showed that leptin treatment *in vitro* significantly enhanced sperm motility and viability. This is consistent with the report of Lange-Consiglio et al. (2016). Lange-Consiglio has concluded that leptin was involved in sperm hyperactivation and capacitation in equine sperm, and improves sperm motility, but for human sperm, Li et al. (2009) suspected that leptin has no significant effect on its motility. The sperm motility is directly associated with ATP production and mitochondrial function. The previous study has been confirmed that leptin increases brain glucose uptake and ATP levels *via* the PI3K/Akt pathway (Zhang et al., 2013). In the current study, we found that leptin treatment for sperms significantly increase MMP. MMP is an ideal parameter that reflects mitochondrial function and mitochondrial energy production ability, thus, it is used to assess the functional integrity of mitochondria. MMP is positively correlated with sperm motility, which has also been confirmed in our study. Agnihotri et al. (2016) suggested that swim-up Sperm of human with highest motility displayed significantly higher MMP, that that of sperm with poor motility at the bottom. Gallo et al. (2021) reported that bovine sperm motility was also positively correlated with MMP.

It is well known that leptin regulates energy metabolism (Magni, Motta & Martini, 2000). We confirmed by immunofluorescence staining that leptin receptors are expressed in the middle of the tail of ram sperm. The middle of the sperm tail is surrounded by a spiral-arranged mitochondrial sheath, and the mitochondrial sheath starts from the beginning of the middle. Surrounding the axoneme and terminating at the terminal ring, mitochondria is cellular energy factory to supply majority of the ATP for sperm motility (Barbagallo et al., 2020). The effects of leptin is mediated by its receptor expressed in the sperms.

In addition to its mobility, leptin treatment also increased the sperm viability compared to the control group by reducing the DFI and ROS. Aquila et al. (2008) reported that leptin activated the anti-apoptotic protein BCL2 *via* its receptor activation to promote the survival of porcine sperm. BCL2, a key protein in survival signaling, was enhanced after leptin exposure, and this effect was diminished by anti-OBR antibodies. Leptin has the ability to attenuate apoptosis among different types of cells, such as osteoblasts, granulosa cells, and islet cells (Almog et al., 2001; Gordeladze et al., 2002), which was consistent with our results that leptin might play a positive role in sperm survival. Another important factor related to the sperm survival is the autophagy. Autophagy can be activated by a number of stimuli, including caloric restriction, oxidative stress, and transient episodes of ischemia and reperfusion (Giricz, Mentzer & Gottlieb, 2012). Its activation can degrade the cellular debris and use them as energy supply. Two well-known signaling pathways including PKA and TOR participate in the process of autophagy (Stephan et al., 2010). Leptin treatment increases the activities of PKA and TOR and also reduces DFI and ROS. As a result, leptin treatment could induce autophagy and improve the energy supply to maintain membrane integrity and energy homeostasis of sperms.

In this study, we have not found that leptin exhibits any effects on the acrosome integrity (AI) and acrosome reaction (AR) of the sperms. This result is consistent with observation of Li et al. (2009). While Lange-Consiglio et al. (2016) demonstrated that the rate of AR increased after leptin treatment in horse sperms. Aquila et al. (2008) speculated that leptin may affect both capacitation and AR through increased cholesterol efflux, protein tyrosine phosphorylation, and acrosome enzyme activity, suggesting its role in the acquisition of fertilization ability of pig sperms. This phenomenon could depend on the concentration of leptin-treated and species specificity.

## Conclusions

This study provides the novel evidence regarding immunolocalization of *OB* and *OBR* in testis, epididymal and ejaculated sperm of rams. A higher mRNA expression of *OB* and *OBR* was observed in epididymal sperm than that of ejaculated sperm, suggesting that when sperm was passing through the epididymis, the surface protein has undergone maturation-related changes, and leptin and its receptors play important physiological roles in spermatogenesis and sperm maturation. In the *in vitro* study, it was found that leptin treatment promotes beneficial effects on spermatozoon quality, enhances sperm motility, viability and MMP, and decreases DFI and ROS compared to the control. Therefore, our study implies that leptin may induce autophagy, decrease external oxidative stress, and maintain membrane integrity and energy homeostasis of ram sperm. These findings may serve as an important pilot study to stimulate the research enthusiasm in this field.

## Supplemental Information

10.7717/peerj.13982/supp-1 Supplemental Information 1 Realtime PCR, RT, WB, serum and seminal plasma levels of leptin, expression of OB and OBR in testis, epididymis, sperm parameters.

10.7717/peerj.13982/supp-2 Supplemental Information 2 Presence of Leptin and the Its Receptor in ram reproductive system and *in vitro* effect of leptin on sperm quality.

## Additional Information and Declarations

### Data Availability

The following information was supplied regarding data availability: The raw data is available in the Supplemental Files.
