# Supplementary figures and images for: Presence of leptin and its receptor in the ram reproductive system and in vitro effect of leptin on sperm quality
Source: PeerJ. 2022 Sep 26;10:e13982. doi: 10.7717/peerj.13982 (PMC9521348; doi:10.7717/peerj.13982)

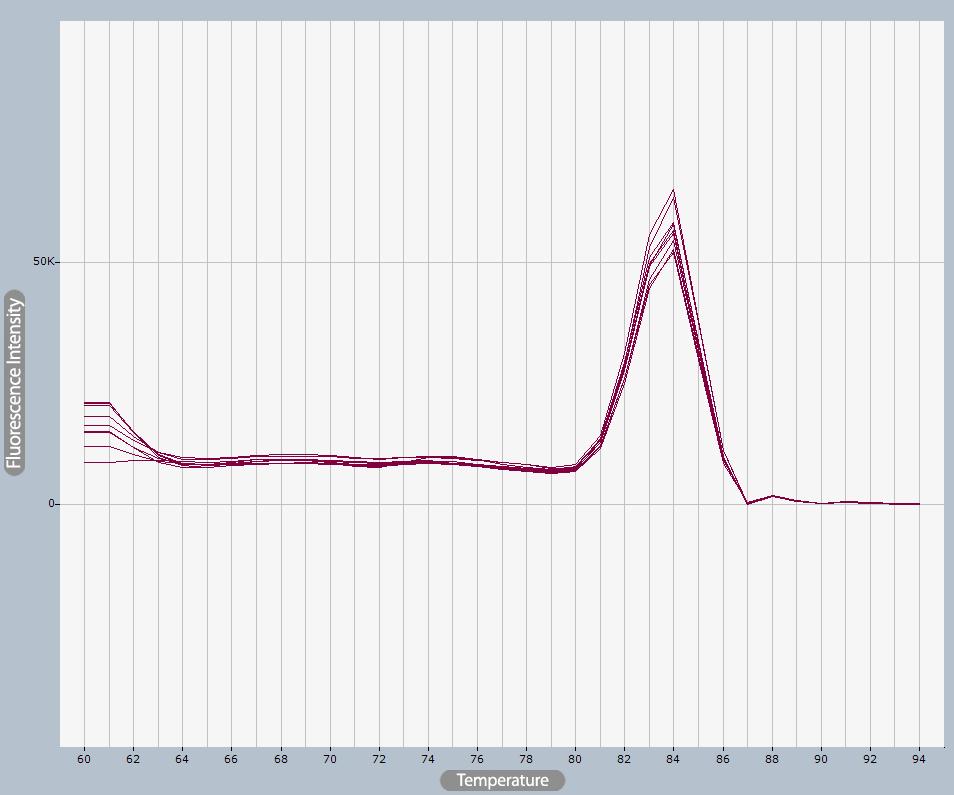

Supplement: Supplemental Information 1 [file peerj-10-13982-s001.zip › supplementary/supplementary/realtime PCR/OB/OB melting.jpg]

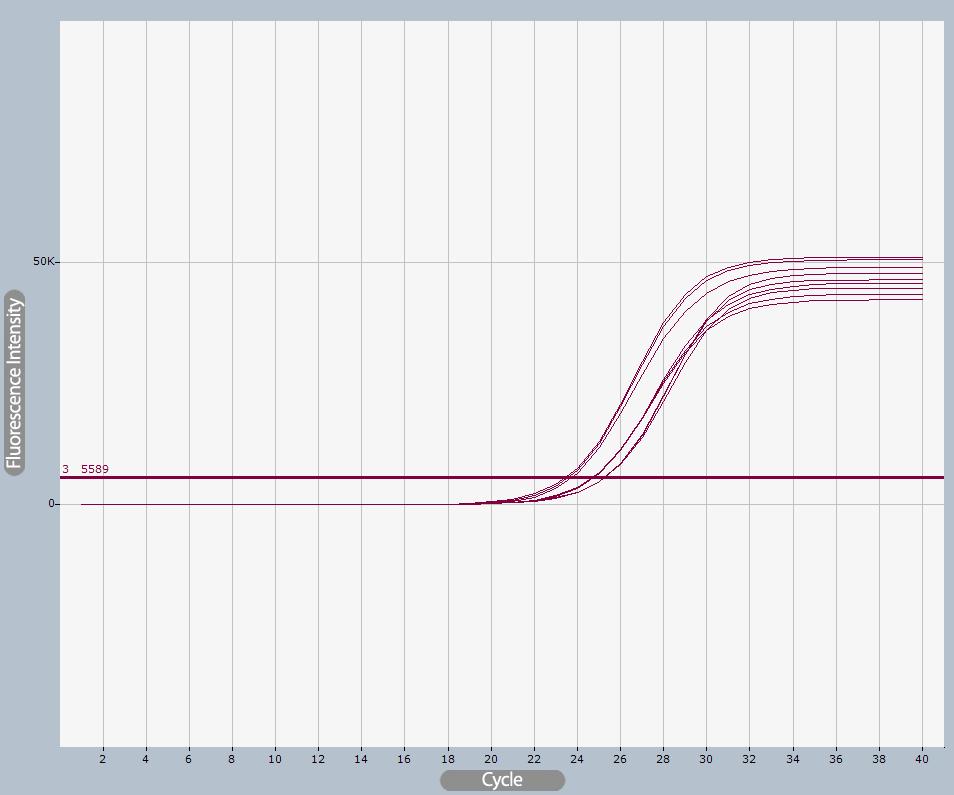

Supplement: Supplemental Information 1 [file peerj-10-13982-s001.zip › supplementary/supplementary/realtime PCR/OB/OB.jpg]

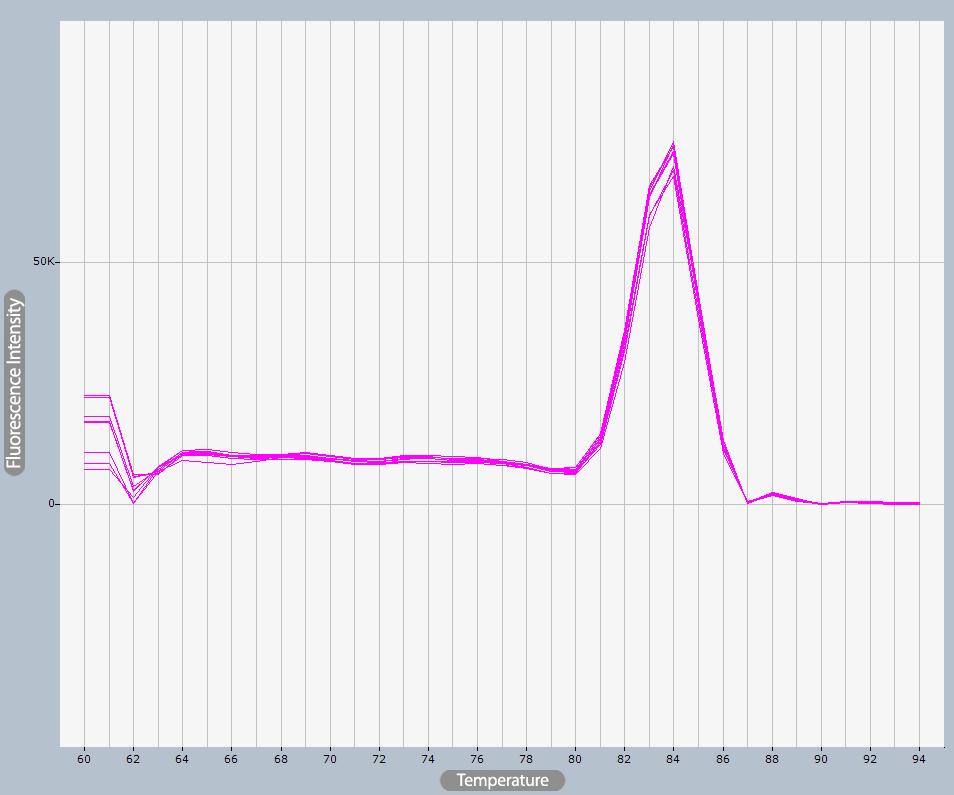

Supplement: Supplemental Information 1 [file peerj-10-13982-s001.zip › supplementary/supplementary/realtime PCR/OB/a┬-actin melting.jpg]

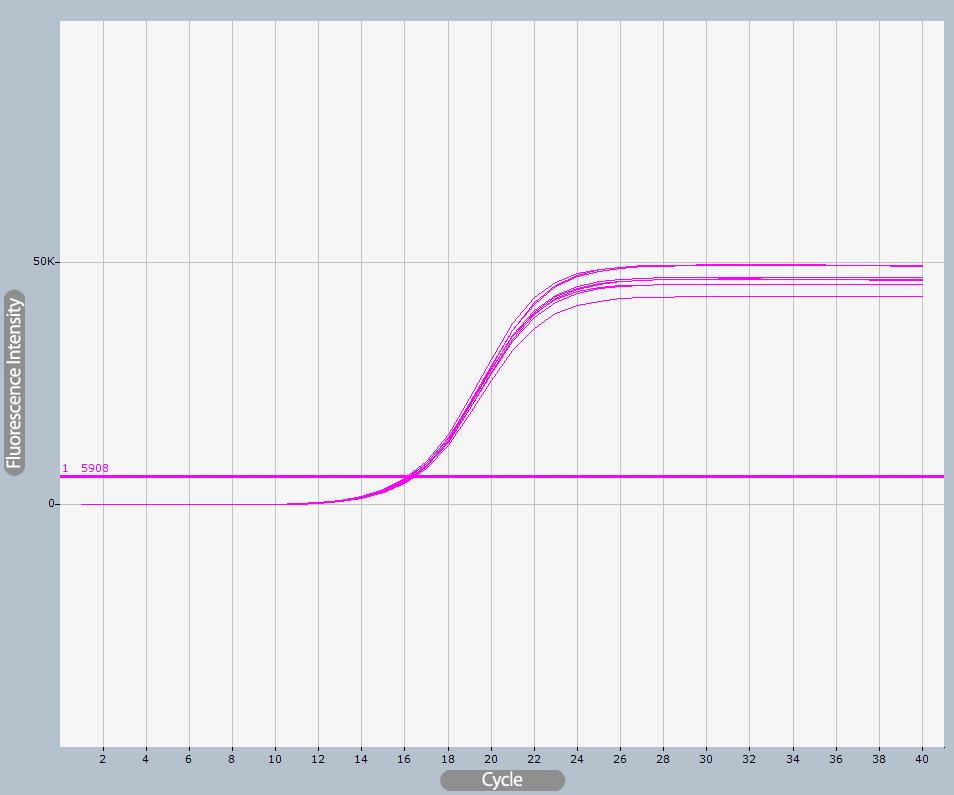

Supplement: Supplemental Information 1 [file peerj-10-13982-s001.zip › supplementary/supplementary/realtime PCR/OB/a┬-actin.jpg]

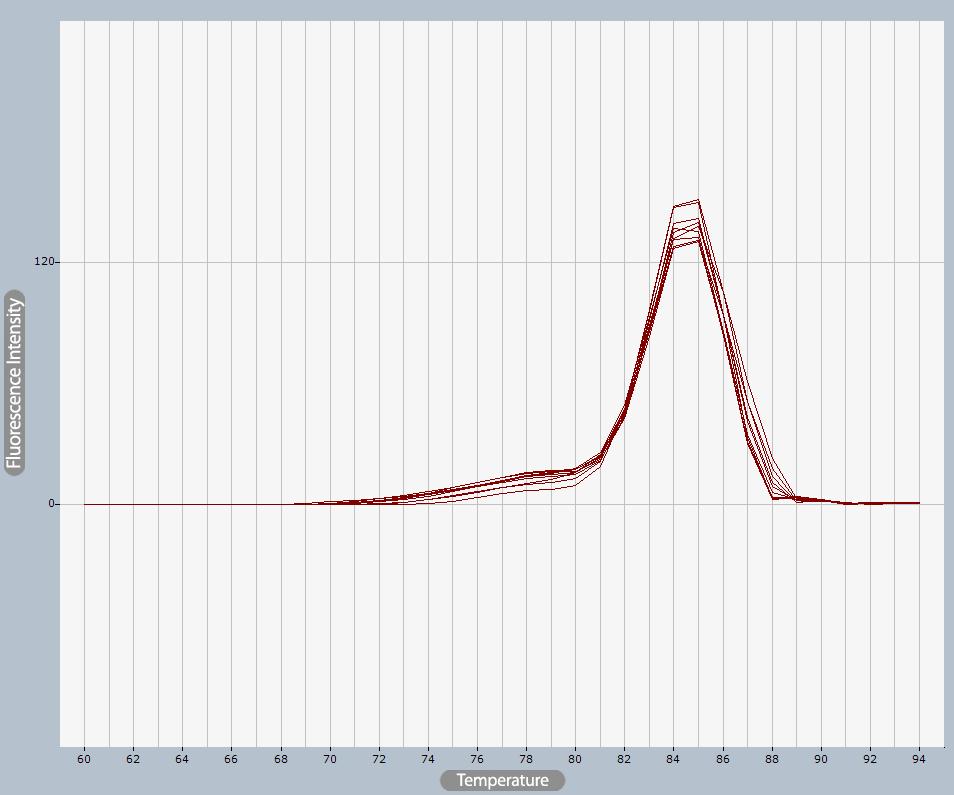

Supplement: Supplemental Information 1 [file peerj-10-13982-s001.zip › supplementary/supplementary/realtime PCR/OBR/OBR melting.jpg]

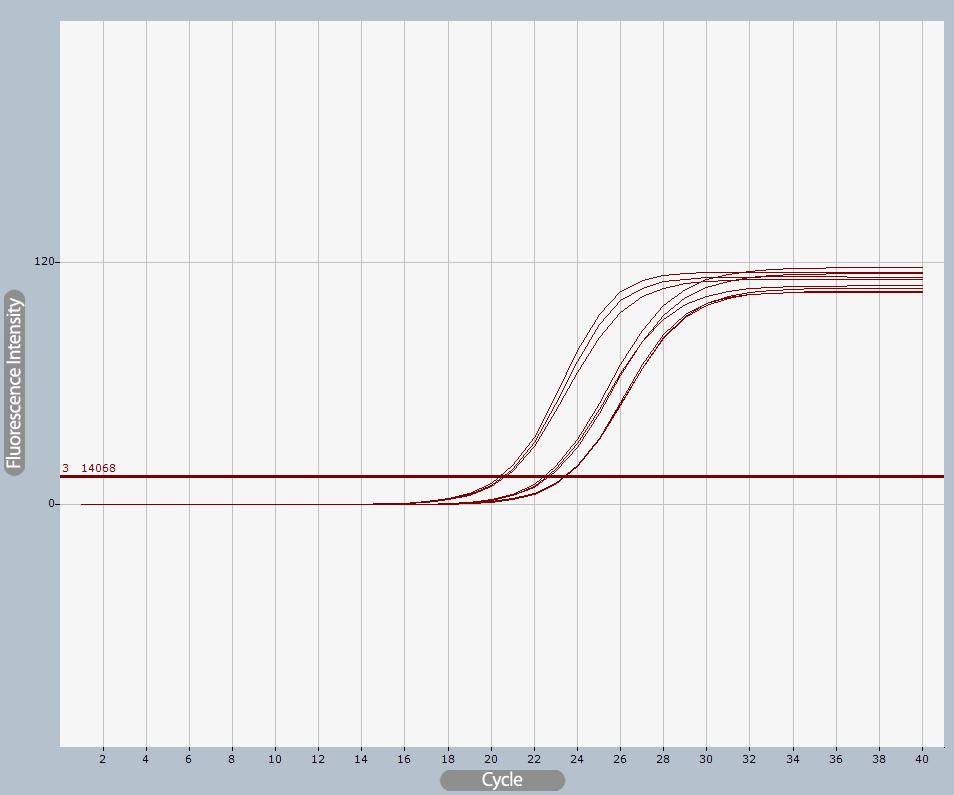

Supplement: Supplemental Information 1 [file peerj-10-13982-s001.zip › supplementary/supplementary/realtime PCR/OBR/OBR.jpg]

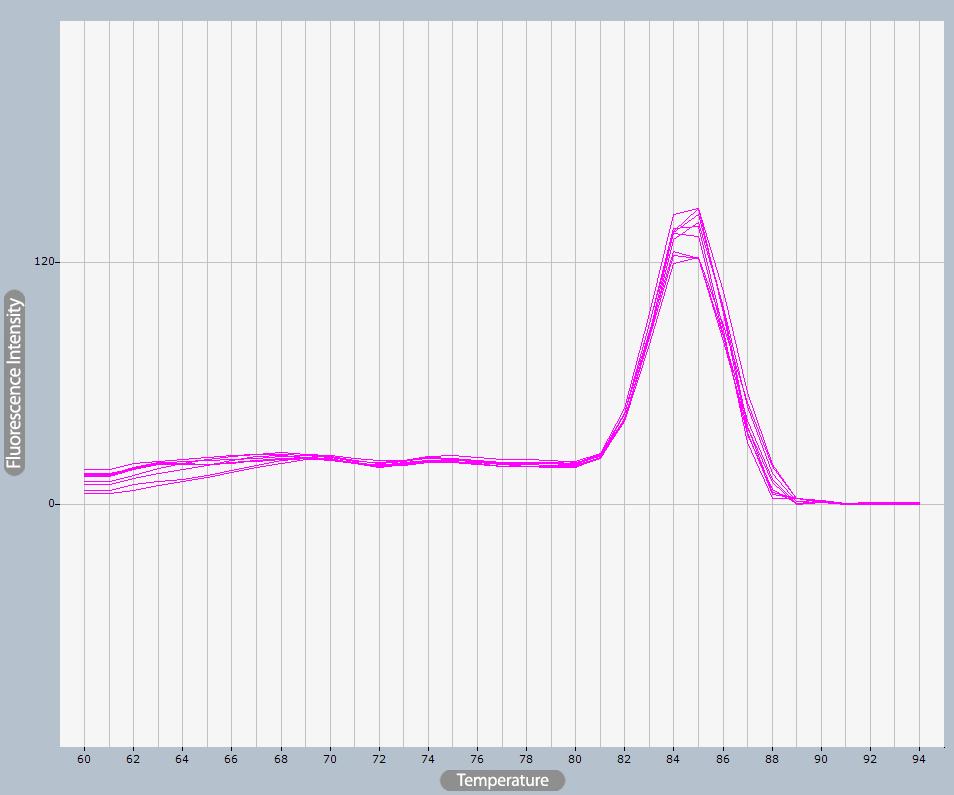

Supplement: Supplemental Information 1 [file peerj-10-13982-s001.zip › supplementary/supplementary/realtime PCR/OBR/a┬-actin melting.jpg]

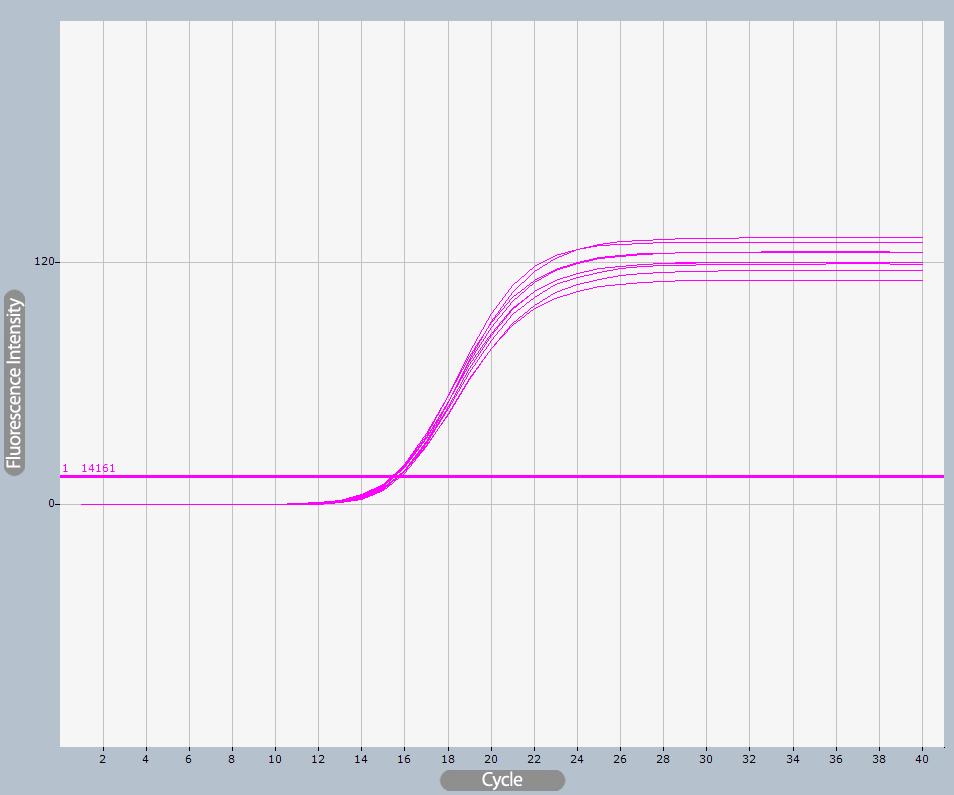

Supplement: Supplemental Information 1 [file peerj-10-13982-s001.zip › supplementary/supplementary/realtime PCR/OBR/a┬-actin.jpg]

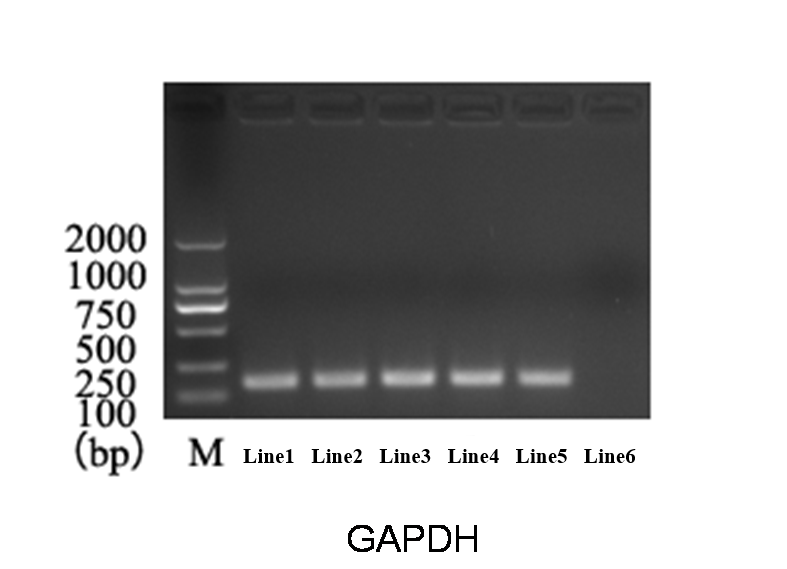

Supplement: Supplemental Information 1 [file peerj-10-13982-s001.zip › supplementary/supplementary/RT/GAPDH-RT1204.tiff]

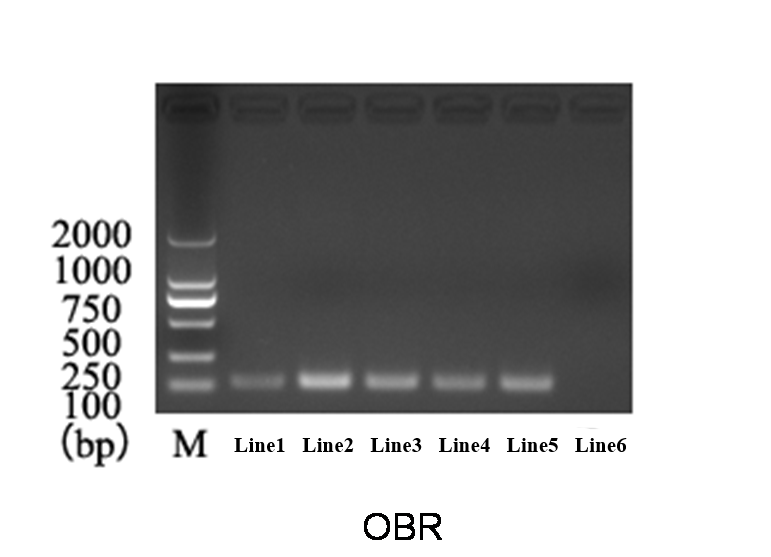

Supplement: Supplemental Information 1 [file peerj-10-13982-s001.zip › supplementary/supplementary/RT/OBR-RT1204.tiff]

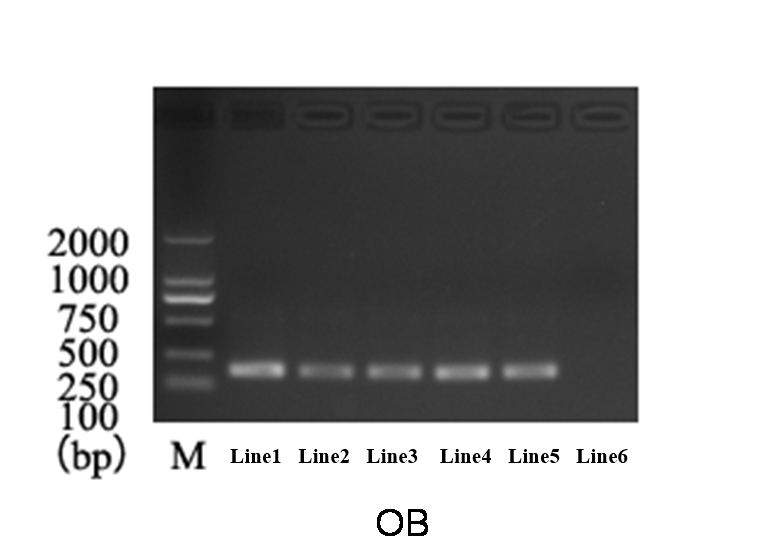

Supplement: Supplemental Information 1 [file peerj-10-13982-s001.zip › supplementary/supplementary/RT/OB-RT.tiff]

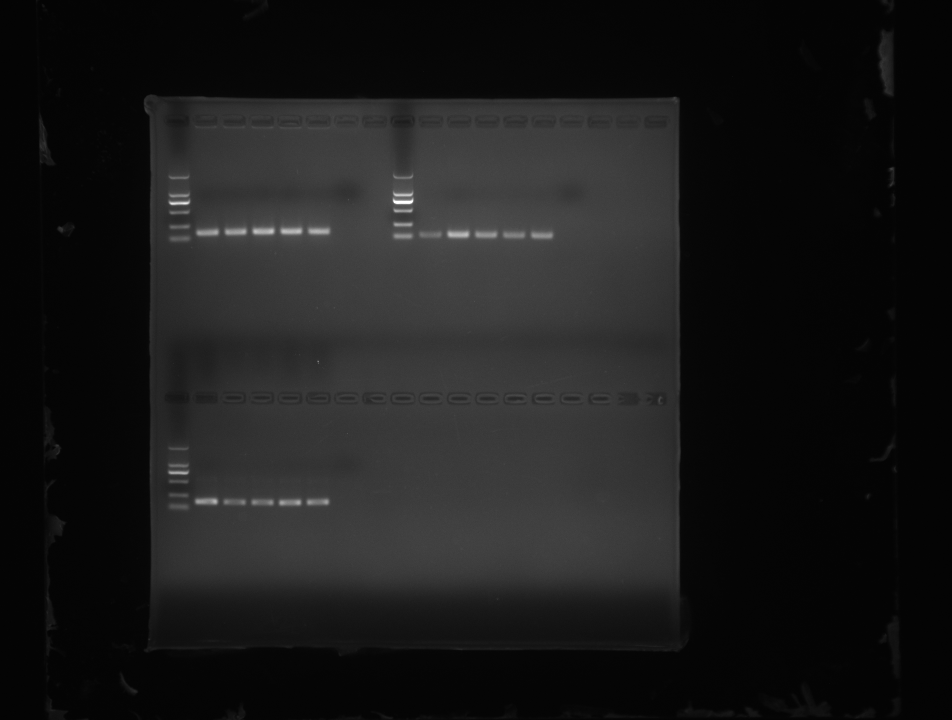

Supplement: Supplemental Information 1 [file peerj-10-13982-s001.zip › supplementary/supplementary/RT/RT╘¡═╝.tif]

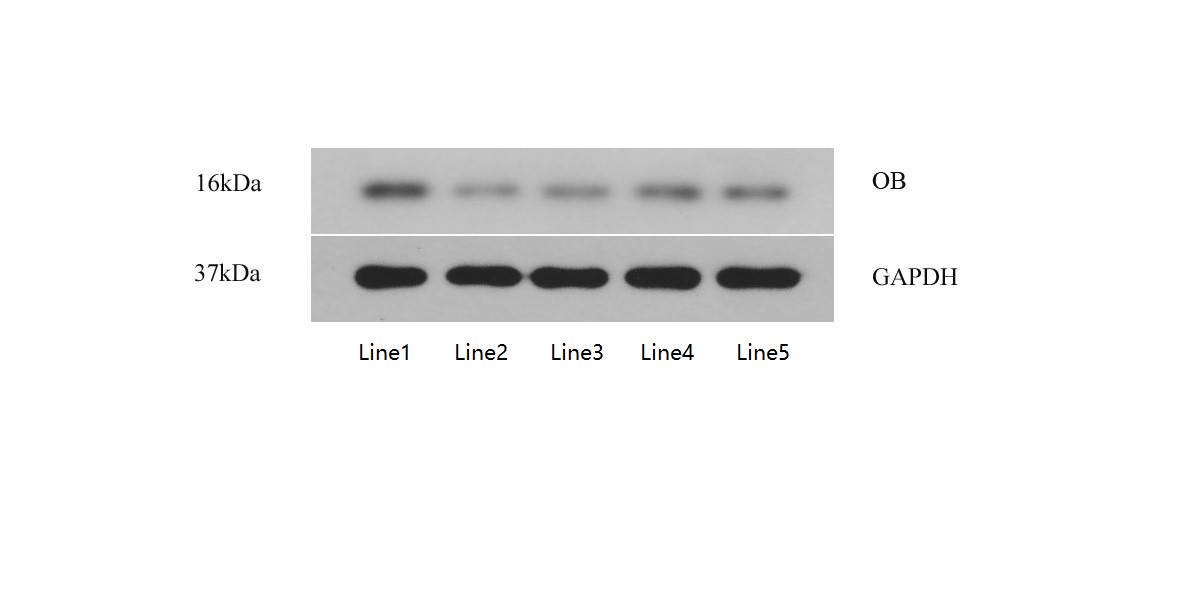

Supplement: Supplemental Information 1 [file peerj-10-13982-s001.zip › supplementary/supplementary/supplementary/WB/113188%23-1+OB111.tif]

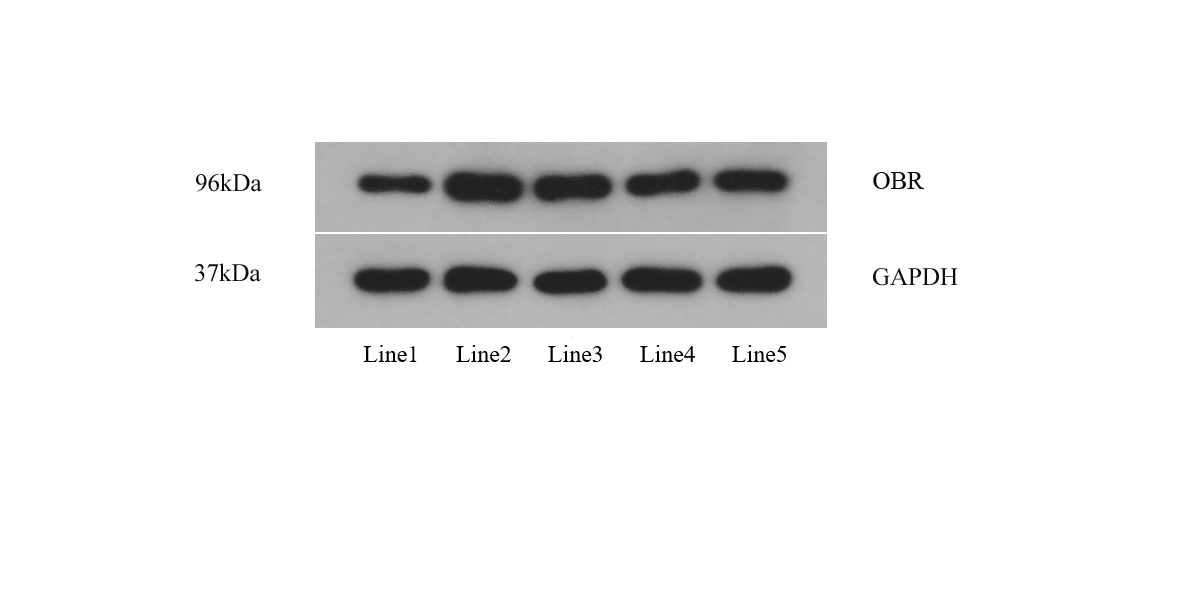

Supplement: Supplemental Information 1 [file peerj-10-13982-s001.zip › supplementary/supplementary/supplementary/WB/113188%23-1+OBR1204.tiff]

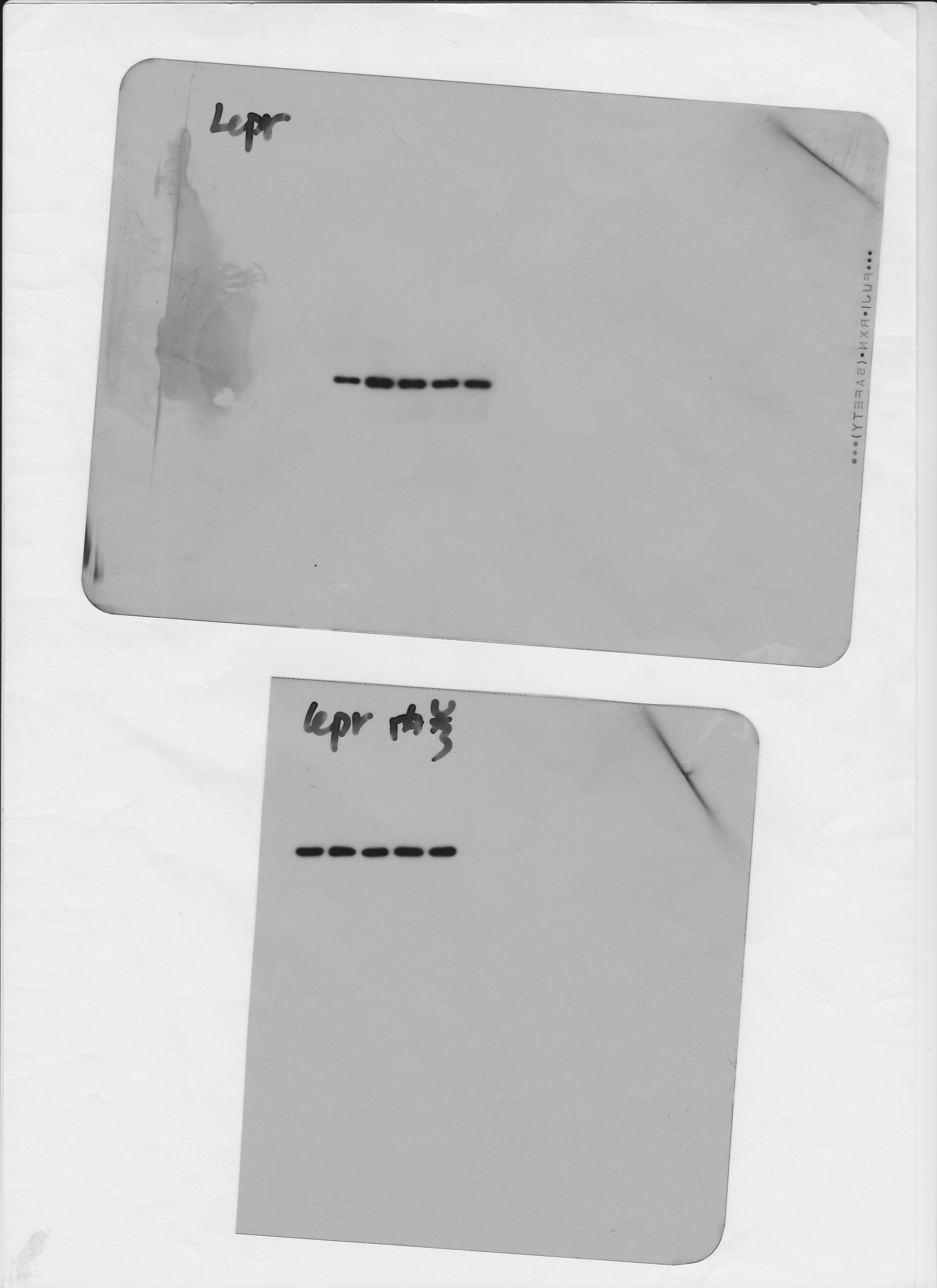

Supplement: Supplemental Information 1 [file peerj-10-13982-s001.zip › supplementary/supplementary/supplementary/WB/IMG_20190925_0029.jpg]

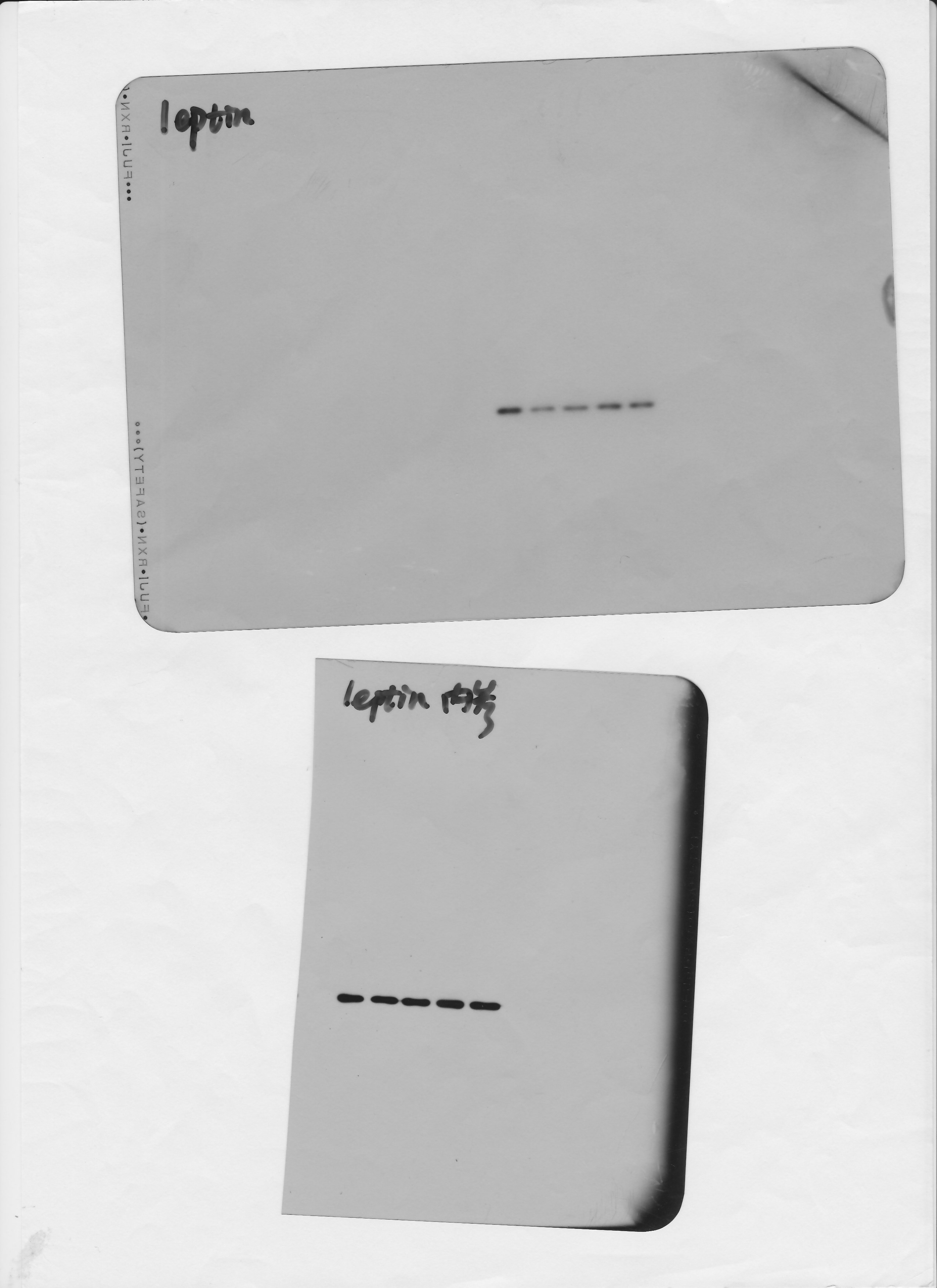

Supplement: Supplemental Information 1 [file peerj-10-13982-s001.zip › supplementary/supplementary/supplementary/WB/IMG_20190925_0030.jpg]
